# Harnessing Self‐Sensitized Scintillation by Supramolecular Engineering of CsPbBr_3_ Nanocrystals in Dense Mesoporous Template Nanospheres

**DOI:** 10.1002/adma.202513469

**Published:** 2025-10-15

**Authors:** Xiaohe Zhou, Matteo L. Zaffalon, Emanuele Mazzola, Andrea Fratelli, Francesco Carulli, Chenger Wang, Mengda He, Francesco Bruni, Saptarshi Chakraborty, Leonardo Poletti, Francesca Rossi, Luca Gironi, Francesco Meinardi, Liang Li, Sergio Brovelli

**Affiliations:** ^1^ Dipartimento di Scienza dei Materiali Università degli Studi di Milano‐Bicocca Via R. Cozzi 55 Milano I‐20125 Italy; ^2^ INFN Sezione di Milano Bicocca Milano I‐20126 Italy; ^3^ Dipartimento di Fisica Università degli Studi di Milano‐Bicocca Piazza della Scienza, 3 Milan I‐20126 Italy; ^4^ School of Environmental Science and Engineering Shanghai Jiao Tong University Shanghai 200240 China; ^5^ IMEM‐CNR Parco Area delle Scienze 37/A Parma 43100 Italy; ^6^ Macao Institute of Materials Science and Engineering (MIMSE) Macau University of Science and Technology Taipa Macao 999078 China

**Keywords:** encapsulation, perovskite nanocrystals, radiation detection, scintillation, supramolecular assembly

## Abstract

Perovskite‐based nanoscintillators, such as CsPbBr_3_ nanocrystals (NCs), are emerging as promising candidates for ionizing radiation detection, thanks to their high emission efficiency, rapid response, and facile synthesis. However, their nanoscale dimensions — smaller than the mean free path of secondary carriers — and relatively low emitter density per unit volume, limited by their high molecular weight and reabsorption losses, restrict efficient secondary carrier conversion and hamper their practical deployment. In this work, a strategy is introduced to enhance scintillation performance by organizing NCs into densely packed domains within porous SiO_2_ mesospheres (MSNs). This engineered architecture achieves up to a 40‐fold increase in radioluminescence intensity compared to colloidal NCs, driven by improved retention and conversion of secondary charges, as corroborated by electron release measurements. This approach offers a promising pathway toward developing next‐generation nanoscintillators with enhanced performance, with potential applications in high‐energy physics, medical imaging, and space technologies.

## Graphical abstract

Perovskite CsPbBr_3_ nanocrystals exhibit bright emission, fast response, and solution processability, but their nanoscale size limits efficient radiation detection. Organizing them into porous SiO_2_ mesospheres enhances radioluminescence up to 40 times, achieving an optimal combination of light yield, fast scintillation, and processability, providing a pathway to high‐performance, versatile nanoscintillators for imaging, space, and high‐energy physics.

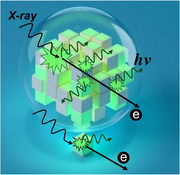

## 1 Introduction

In recent years, the need to overcome the performance and scalability limitations of current ionizing radiation detectors^[^ ^1^, ^2^ ^]^ has stimulated a growing interest in new generation scintillator materials that offer innovative platforms to address technological challenges in areas such as medical and high energy physics,^[^ ^3^, ^4^ ^]^ space exploration,^[^ ^5^ ^]^ and radiometric imaging.^[^ ^6^, ^7^ ^]^ These include so‐called nanoscintillators,^[^ ^3^, ^8^, ^9^ ^]^ which consist of chemically produced semiconductor nanoparticles that offer a combination of the high average atomic number *Z* required for interaction with ionizing radiation^[^ ^3^, ^10^, ^11^, ^12^ ^]^ typical of expensive inorganic scintillator crystals, intense and ultrafast scintillation promoted by the formation of band‐edge multi‐excitonic states,^[^ ^12^, ^13^, ^14^ ^]^ radiation stability,^[^ ^15^ ^]^ and production scalability comparable or superior to traditional aromatic chromophores used in plastic scintillators.^[^ ^16^ ^]^ Among these, lead halide perovskite nanocrystals (NCs) of the general formula CsPbX_3_ (where X is Cl, Br or I) have rapidly become the archetypal material,^[^ ^17^ ^]^ further favored by their defect tolerance,^[^ ^18^, ^19^, ^20^ ^]^ wide chromatic tunability,^[^ ^21^, ^22^ ^]^ radiation hardness^[^ ^15^ ^]^ which is favored by the mobility of the ionic lattice, that promotes self‐healing.^[^ ^23^, ^24^ ^]^ They also feature fast scintillation dynamics^[^ ^25^, ^26^ ^]^ and are easily synthesized in large quantities at room temperature.^[^ ^16^, ^27^, ^28^ ^]^ An increasing number of studies have therefore been devoted to optimising their optical properties,^[^ ^29^, ^30^, ^31^, ^32^, ^33^, ^34^ ^]^ their compatibility in plastic matrices,^[^ ^12^, ^13^, ^35^, ^36^, ^37^, ^38^ ^]^ including scintillating polymers,^[^ ^13^, ^39^ ^]^ and to obtain a detailed understanding of the mechanism of scintillation at the nanoscale, both at the level of isolated NCs^[^ ^26^, ^40^ ^]^ and at the level of nanocomposites.^[^ ^13^, ^14^, ^41^ ^]^ Based on these advances, important guidelines are now available for the design of CsPbX_3_ NC‐based scintillators that maximise the speed and efficiency of the scintillation process.

However, important challenges remain in making this new class of nanocomposite scintillators applicable in real‐world contexts. Indeed, a key aspect of nanocomposite scintillators is that the particle size is significantly smaller than the average free path of secondary charges (e.g., electrons) produced as a result of primary interactions with ionising radiation,^[^ ^41^, ^42^, ^43^ ^]^ which causes the electromagnetic shower produced by an NC to escape from the source particle.^[^ ^12^, ^26^, ^41^ ^]^ This favors their use as sensitizers of secondary emitters through both their emission and their shower (still, however, lowering the scintillation speed) but limits the ability of matrix‐dispersed NCs to retain and convert energy into ultrafast light pulses. As in the case of conventional plastic scintillators, it is therefore important to design strategies also capable of trapping and converting secondary charges. In nanocomposite scintillators based on heavy NCs, however, this need is met by a seemingly intrinsic limitation represented by their high *Z*, which gives average molecular weights of many orders of magnitude higher than those of conventional organic dyes (200–4000 kg mmol^−1^ for 3–8 nm NCs vs 0.2–0.5 kg mmol^−1^). In X‐ray imaging screens, this is actually advantageous because it allows high stopping power from a few layers of NCs, minimising scattering while improving image quality.^[^ ^44^, ^45^ ^]^ In massive devices, on the other hand, it means that for the same mass percentage, the number of NCs in a plastic matrix is orders of magnitude lower than in their organic counterparts, with a correspondingly greater interparticle spacing. Consequently, although nanocomposite scintillators exhibit scintillation efficiencies competitive with even commercial plastic scintillators when irradiated with soft radiation (favored by their greater primary interaction capacity),^[^ ^12^, ^38^, ^43^ ^]^ their application as high‐energy radiation detectors becomes increasingly problematic. This is precisely because of the increasing weight of secondary processes relative to primary ones as the energy of the incident radiation increases (reducing the fraction of energy deposited within a single NC) and the difficulty of exploiting them effectively. From a practical point of view, achieving emitter densities, in terms of NC cm^−3^, comparable to those of molecular scintillators (≈1wt.% corresponding to ≈10^20^ molecule cm^−3^), by proportionally increasing the NC load, would entail weight fractions incompatible with the chemistry of real plastic nanocomposites (up to over 100% of the volume) and would entail high optical losses due to reabsorption and scattering of the scintillation light. As a result, effective collection and conversion of secondary carriers is an open challenge in NC‐based scintillators.

In this context, recent results on polyvinyl toluene nanocomposites with high loading of CsPbBr_3_ NCs showed a substantial increase in scintillation yield as a result of partial phase segregation of NCs in high particle density domains.^[^ ^39^ ^]^ This local densification, albeit uncontrolled, resulted in a seemingly quadratic growth of scintillation intensity with NC loading compared to a linear growth of the same in similar solutions, leading to a 10‐fold increase in scintillation yield at high loadings and suggesting the possibility of exploiting both direct intra‐NC interactions and an inter‐NC self‐sensitisation process via the secondary shower by supramolecular control of the local spatial distribution of NC in the matrix. Such a scheme has not yet been demonstrated and might provide a viable strategy for advancing nanocomposite scintillators.

**Figure 1:**
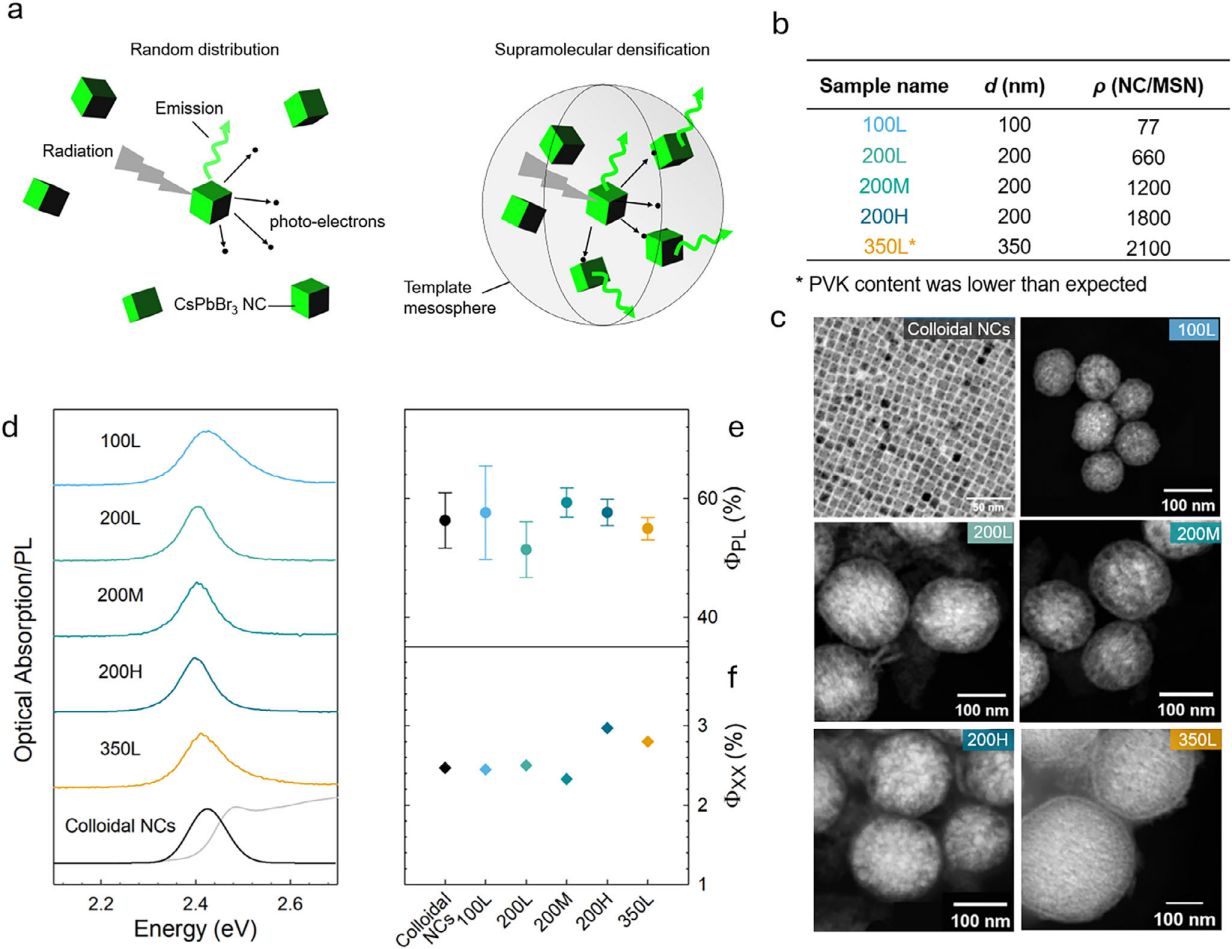
Concept of locally densified NC scintillators and controlled model system. a) Scintillation mechanism in isolated NCs versus engineered densified structures. b) Sample description with MSN diameter (*d*) and NC density per MSN. c) TEM images of NC‐MSNs of sizes *d* = 100, 200, and 350 nm. d). Optical absorption (gray line), PL spectra of colloidal NC (black line) and NC‐MSN (colored lines) dispersed in toluene. Excitation energy *E_ex_* = 3.05 eV. e), PL quantum yield (*Φ_PL_*) and f) biexciton quantum yield (*φ_XX_*) of colloidal NCs and NC‐MSNs. *φ_XX_* is extracted from the fitted single exciton and biexciton components of TA dynamics at their corresponding 1S bleach maximum using the successive subtraction method.^[^ ^1^, ^2^ ^]^

Here, we aim to contribute to this effort by investigating the effectiveness of engineering the arrangement of scintillating NCs into dense domains that locally mimic the inter‐emitter spacing of organic scintillators, thus allowing us to exploit the advantages of the high *Z* of heavy NCs while also converting secondary charges (see sketch in **Figure** **1**a). To this end, we have exploited the possibility of synthesising CsPbBr_3_ NCs inside the pores of mesoporous SiO_2_ nanospheres (MSNs),^[^ ^46^, ^47^ ^]^ which act both as a templating agent for the realisation of distinct and processable supramolecular units containing isolated NCs in a controlled number and distribution, and as a protective shell that stabilises the host particles against external agents (H_2_O, environmental pollutants) and prevents the release of Pb ions into the environment,^[^ ^46^, ^48^ ^]^ potentially allowing their use also in biological contexts.^[^ ^49^, ^50^, ^51^ ^]^ Radiometric experiments on model samples composed of NCs of the same size and specifically designed to exhibit identical optical properties in both the excitonic (*X*) and biexcitonic (*XX*) regimes show that, for the same total concentration of NCs in solution, their arrangement in mesospheres gives rise to a substantially greater radioluminescence (RL) than their colloidal counterparts. The intensity of the RL increases progressively with the size of the MSNs for the same density (NCs cm^−3^) or their density at constant MSN size, resulting in up to a 40‐times higher RL yield compared to standard colloidal suspensions. Consistent with enhanced density of photoelectrons inside the MSNs following ionizing excitation, the increase in scintillation is accompanied by a progressive growth in the ultrafast contribution by charged excitons (or trions, X*) to the emission kinetics, which is typically absent in colloidal suspensions or dilute composites,^[^ ^26^, ^38^ ^]^ opening to the possibility of further improving the timing capability of individual NCs by their engineered assembly. The direct correlation between the increase in scintillation properties and the conversion of secondary charges within the NC containing MSNs is further independently demonstrated by the experimental measurement of singlet oxygen (^1^O_2_) generation in aqueous solution prompted by the electric shower released by the particles following X‐ray excitation, which anticorrelates quantitatively with the RL intensity trend. Finally, Monte Carlo simulations with Geant4 show that the amount of deposited energy increases proportionally to the degree of local densification and provide a deeper insight into the self‐sensing mechanism as a function of material parameters. These results demonstrate a useful paradigm for the design of nanoscintillators and open up the possibility of realising metasolids that best exploit the advantages of dimensionally confined materials for radiation sensing, also potentially expanding the applicability of recent strategies for NCs embedding inside metal–organic frameworks (MOF),^[^ ^47^ ^]^ amorphous network structures,^[^ ^52^ ^]^ and mesoporous nanoparticles^[^ ^46^, ^53^ ^]^ that have been employed to preserve the optical properties in harsh environments.

## 2 Results and Discussion

The scintillation properties of CsPbBr_3_ NCs are determined by structural and photophysical parameters that jointly determine the behavior in the *X* and *XX* regimes, both of which are present under ionising excitation.^[^ ^13^, ^26^ ^]^ In particular, size determines the average energy deposited in an NC and hence the average excitonic occupancy (*N*) produced following a multi‐exciton generation process^[^ ^3^, ^12^, ^26^, ^54^ ^]^ and, via volume scaling,^[^ ^55^ ^]^ the efficiency of the Auger recombination process, which determines the *XX* and X* recombination yield. The photoluminescence (PL) quantum yield (Φ_PL_) determines the contribution of *X* (generated directly or as a result of *XX* recombination) to the total emission. The relationship between scintillation intensity and dynamics and these photophysical parameters follows a complex dependence,^[^ ^26^ ^]^ which weakens a performance comparison by a posteriori corrections of experimental data. For this reason, and in order to accurately assess the local densification effects of NCs, CsPbBr_3_ NC‐based samples with nearly identical optical and structural properties were realized. First, we synthesized a series of uniform MSNs following a modified protocol, which were then used as nano‐templates for the confined growth of CsPbBr_3_ NCs.^[^ ^47^ ^]^ The MSNs exhibited uniform sizes of *d* = 100, 200, and 350 nm (Figure S1, Supporting Information) with long‐range ordered pore structures with comparable pore sizes and proportionally larger specific surface areas. The templates were then soaked in the solution of the precursor salts (CsBr and PbBr_2_) and then dried at 80 °C to synthesise CsPbBr_3_ NCs in their inner pores. The resulting mixture was placed in a furnace and heated to 600 °C. The cooled samples were washed with ultrapure water and dried to obtain the final product. In order to study the effect of the size of artificially densified NC domains, a set of NC‐loaded MSNs (hereafter indicated as NC‐MSNs) of the three different sizes was prepared by keeping the density of CsPbBr_3_ NCs per MSN constant at 10 wt.%, corresponding to *ρ* = 77, 660, and 2100 NCs MSN^−1^, respectively. As shown in Figure 1b, which reports the results of the chemical analysis of the samples, the actual CsPbBr_3_ content of the *d* = 350 nm NC‐MSNs was lower than the nominally expected value (*ρ* = 3300 NCs MSN^−1^), probably due to the difficulty of the reagents to reach the innermost particle pores, resulting in impregnation of only the outermost nanosphere shell. However, this sample was kept in the set throughout the study because, as shown below, its off‐trend behavior in both RL yield and electron retention capability actually indirectly confirms the size dependence of the others (vide infra). For the set of samples with increasing density of NCs, *d* = 200 nm MSNs were used as templates, and the loading of NC concentration was progressively increased from 10 wt.% (comparable to the *d* = 100 nm NC‐MSNs) to 20 and 30 wt.% to obtain densities of *ρ* = 1200 and 1800 NC MSNs^−1^. Finally, a sample of colloidal CsPbBr_3_ NCs of comparable size (8 ± 0.9 nm) was prepared according to a literature protocol.^[^ ^56^ ^]^

**Figure 2:**
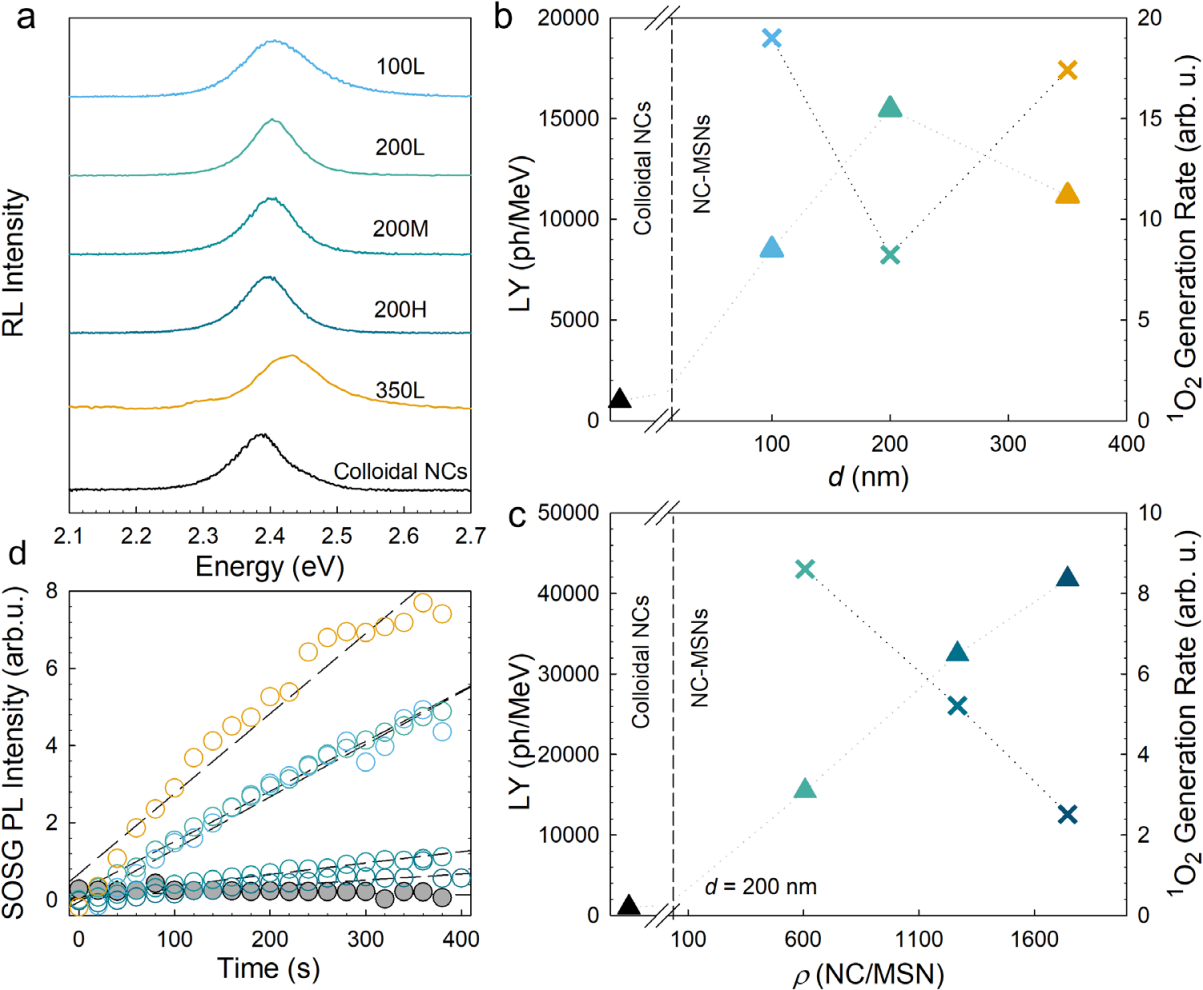
Radioluminescence (RL) and electron release from supramolecularly densified nanoscintillators. a) RL spectra of NC‐MSNs (colored lines) and analogous colloidal CsPbBr_3_ NCs reference (black line) dispersed in dodecane. The total CsPbBr_3_ contents in each sample solution are maintained at 0.1wt.%. The same color code is applied to all panels. b) Light yield of NC‐MSN normalized to colloidal NCs, overlayed with ^1^O_2_ generation rate as an indicator of released electrons as a function of increasing MSN size (constant density as shown in Figure 1b). c) LY/^1^O_2_ generation rate as a function of number of NCs per MSN (*d* = 200 nm). The ^1^O_2_ generation rates are extracted from the slope of the respective growth of SOSG intensity (excited at 473nm) normalized by the initial SOSG intensity of pristine MSN (black circles) and NC‐MSNs (colored circles) with increasing X‐ray exposure time shown in “d”.

Figure 1c shows the transmission electron microscopy images of the investigated NC‐MSNs together with the corresponding colloidal suspension, showing distinct crystalline CsPbBr_3_ NCs with a cubic structure (as confirmed by X‐ray diffraction measurements, Figure S2, Supporting Information) and an average size of 8 ± 0.9 nm within the MSNs. The PL spectra of the samples (Figure 1d) show the typical excitonic emission peak of CsPbBr_3_ NCs at ≈2.4 eV, confirming the comparable size of the embedded NCs. TheΦ_PL_ of all systems dispersed in toluene was measured in an integrating sphere and shows an essentially constant value within the error bars of ≈55 ± 6% (Figure 1e). To investigate the local optical properties of the NC‐MSNs, cathodoluminescence (CL) measurements were performed on all samples. Figure S3 (Supporting Information) shows CL maps acquired using an electron beam current of 300 pA and an accelerating voltage of 5 kV. The corresponding spectra are shown in Figure S4 (Supporting Information); histograms showing the CL intensity per unit area of the analysed particles are reported in Figure S5 (Supporting Information). For the spheres with diameters *d* = 100 nm, the number of NCs in each sphere (77 as indicated in Figure 1b) is too low to allow successful CL mapping. CL images of the *d* = 200 nm samples at increasing concentrations clearly show a corresponding increase in luminescence, particularly between the 200L and 200M samples. Regarding the 350 nm MSNs, a significant inhomogeneity in NC incorporation and a reduction in CL mean intensity compared to the 200 nm MSNs were observed. This is consistent with the decrease in RL intensity reported in **Figure** **2**.

As mentioned above, the scintillation mechanism in NCs is strongly dependent on the multi‐exciton photophysics. Therefore, we performed transient transmission measurements as a function of excitation fluence to assess the *XX* yield. This was done following the standard approach in carrier dynamics studies in NCs,^[^ ^57^ ^]^ i.e., by measuring the bleach dynamics of the 1S absorption feature at increasing fluence (see Figures S6 and S7, Supporting Information) and subtracting the lowest fluence dynamics due to *X* decay from the other to extract the *XX* decay rate, *k_XX_*. The *XX* efficiency was then estimated under the common assumption that the low fluence dynamics correspond to *X* radiative decay (with rate $k_{X}^{R}$) and considering the statistical relation $k_{XX}^{R}=4k_{X}^{R}$, so that the *XX* yield can be written as $\Phi_{XX}=k_{XX}^{R}\;/k_{XX}$.^[^ ^57^ ^]^ The resulting Φ_*XX*_ values for the whole sample set are shown in Figure 1f and again show a constant value of ≈2.5%, in agreement with previous reports on CsPbBr_3_ NCs of comparable size.^[^ ^58^, ^59^ ^]^

After confirming the validity of the model sample set and the consistency of the respective optical properties as the degree of local organization of the NCs was varied, we proceeded with a quantitative comparison of their scintillation under X‐ray excitation from a tungsten cathode (average energy 10 keV, 20 mA). For this purpose, dispersions with the same average concentration of CsPbBr_3_ NCs were used, as confirmed by optical absorption measurements (Figure S8, Supporting Information). This implies that the content of emitting species is constant (i.e., CsPbBr_3_ NCs), while the number of NC‐MSNs in solution changes on a case‐by‐case basis depending on their NCs content. To eliminate possible artifacts due to different outcoupling of the scintillation light and to avoid spurious contributions from the solvent, all NC‐MSNs samples were surface functionalized with octadecyltrichlorosilane (OTS) to maximize their solubility and redispersed in octane, which has no scintillation of its own. Colloidal NCs did not require surface functionalization. Finally, to show differences due to local densification of the NCs by the template, the total concentration of CsPbBr_3_ was kept low so that inter‐NC interactions in the colloidal solution were negligible. The RL spectra are shown in Figure 2a and show agreement with the corresponding PL and CL, confirming the absence of parasite emission due to MSNs or defect states. RL measurements measured under continuous X‐ray irradiation confirm the stability of MSN‐CsPbBr_3_ NCs up to 840 Gy doses (Figure S11, Supporting Information). Given the complete similarity of the spectral features, the corresponding scintillation yields measured under identical excitation and collection conditions show substantial differences, confirming the hypothesized self‐sensitization effect due to the spatial organization imposed by the MSN templates. Specifically, Figure 2b shows the light yield (LY, defined as the number of emitted photons per unit energy deposited) of the investigated systems (300 µL, 1%wt. in a 0.5 cm high cylindrical cuvette) measured in the same excitation and collection geometry at room temperature as a commercial plastic scintillator EJ‐276D (LY = 8600 photons MeV^−1^)^[^ ^60^ ^]^ of the same volume (in all cases resulting in essentially complete deposition of the incident X‐ray excitation). Although the concentration of CsPbBr_3_ NCs in solution was constant, increasing the size of the mesosphere gradually enhanced the LY, reaching a 15‐fold enhancement in the *d* = 200 nm NC‐MSNs compared to the respective colloidal solution. We note that the use of larger template MSNs (*d* = 350 nm) led to a decrease in LY, consistent with the lower atomic content shown in Figure 1b, resulting in greater electronic losses. This is further confirmed by the electron release measurements and time‐resolved RL experiments discussed below (vide infra). Increasing the NC density within the *d* = 200 nm MSNs—for which effective embedding of large amounts of CsPbBr_3_ was possible—resulted in further progressively higher LY, yielding a total enhancement of 40 times with respect to a comparable amount of colloidal NCs (Figure 2c). The dramatic enhancement of RL in NC‐MSNs agrees well with recent theoretical results by Villa et al. that revealed nearly complete deposition of energy inside SiO_2_ nanospheres following X‐ray excitation, which, in the current case, results in stronger NC excitation.^[^ ^61^ ^]^ We emphasize that the optical density due to CsPbBr_3_ NCs is constant for all samples studied, and thus these experiments validate the concept of engineering the local distribution of NCs to enhance their scintillation yield without introducing additional loss due to reabsorption that would be direct consequence of enhancing the average NC concentration, which is currently an unresolved limitation in the use of NCs with excitonic emission in scintillation.^[^ ^39^ ^]^ Consistently, side‐by‐side RL measurements on film samples of the same colloidal NCs and NC‐MSNs (containing the same amount of CsPbBr_3_ as for the solution study in Figure 2) show comparable intensity across the sample set (Figure S13, Supporting Information), further confirming that the enhancement effect is due to the densification and consequent improved collection of secondary carriers. As a result of such a dramatic improvement in RL with respect to colloidal NCs, the LY reaches 40 000 ± 1000 photons MeV^−1^, which is also consistent with the observed enhancement with respect to reported results on diluted CsPbBr_3_ NCs solutions and composites.^[^ ^26^, ^39^ ^]^ Please refer to Table S2 (Supporting Information) for a comparative overview of LY values reported in the literature for CsPbBr_3_ NCs and related structures. This comparison underscores that supramolecular densification achieves the highest LY reported for colloidal suspensions and exceeds the performance of thick CsPbBr_3_‐based coatings, offering the most advantageous combination of high efficiency, fast decay time, and material processability reported to date. It is nonetheless important to emphasize that the LY of NC‐based scintillators – particularly in light of the present results – must be interpreted with caution. The measured LY is highly sensitive to sample‐specific characteristics, including NC concentration (in solution), thickness (for film‐based samples), optical haziness (especially in micropowders or coatings), as well as the efficiency of secondary carrier conversion. The latter decreases with increasing irradiation energy, leading to pronounced energy dependence. Consequently, both in general and in the present study, a significant nonlinearity of LY as a function of irradiation energy should be anticipated.

To strengthen the attribution of the observed increase in RL intensity to increased conversion of secondary charges into scintillation light, we independently measured electron release following ionizing interaction. To do this, we used a fluorescent singlet oxygen sensor (SOSG),^[^ ^62^ ^]^ commonly used in radiotherapy studies, to monitor in situ the evolution of ^1^O_2_ generated following electron release by X‐ray sensitizers under irradiation. In its non‐oxidized form, SOSG is non‐emissive, while its endoperoxide derivative, formed as a result of oxidation by ^1^O_2_, exhibits a PL at 530 nm (Figure S9, Supporting Information), the intensity of which is commonly used to quantify the amount of ^1^O_2_ species generated during X‐ray irradiation, which in turn is proportional to the release of electrons into solution. In this study, SOSG was mixed with NC‐MSN in aqueous solution and excited by a laser at 473 nm; each scan lasted 10 min of continuous exposure from the same X‐ray source used for the RL measurements. Figure 2d reports the time evolution of the SOSG PL intensity using NC‐MSNs as well as empty MSN templates and a bare water solution. Consistent with previous results,^[^ ^62^ ^]^ the introduction of heavy NC‐MSNs largely increases the SOSG emission, which further suggests their potential use as luminescent X‐ray sensitizers in radiotherapy. Most relevant to this work, the ^1^O_2_ generation rates, extracted as the slope of the curves in Figure 2d and reported as crosses in Figure 2b,c, anticorrelate remarkably well with the respective RL intensities, thus independently supporting the picture that the stronger RL intensities are in fact associated to better retention and conversion of secondary electrons, once again in agreement with previous reports.^[^ ^61^ ^]^

**Figure 3:**
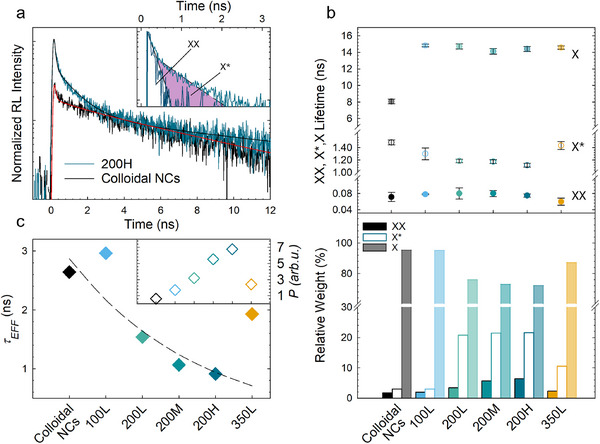
Dynamic effects of supramolecular densification. a) Time‐resolved RL of colloidal NCs (black line) and 200H (dark green line), normalized to single exciton tail. Inset: X* (colored line) and XX(black line) dynamics of 200H are explored at a fast‐time scale (time window 0–3ns) by subtracting the contribution from the single exciton, showing the charged exciton following the successive subtraction method.^[^ ^1^, ^2^ ^]^ The shaded area highlights the contribution from X* in the NC‐MSN. b) Decay lifetimes (upper panel) and relative weight (lower panel) of X (light colors), X* (hollow) and XX (dark color) contributions of colloidal NCs and MSN samples. c) Effective lifetime *τ_EFF_* of colloidal NCs and MSN. Inset: Pulse quality factor *P* across the sample set following the same color code as the main plot.

The local organization of NCs into MSNs, in addition to increasing the scintillation yield, also has a relevant impact on the RL kinetics, providing further independent evidence for the increased electron density within the NC‐MSNs. As demonstrated in **Figure** **3**a, which compares the RL time decays under X‐pulsed excitation of colloidal NCs and NC‐MSNs (*d* = 200 nm, normalized to their long time tails to emphasize the different ultrafast contributions, the whole set of data is reported on Figure S10, Supporting Information), the decay of colloidal NCs is dominated by a 10 ns component attributed to *X* decay and a relatively small ultrafast contribution due to the combination of X* and *XX* emission.^[^ ^26^ ^]^ In accordance with the literature, the decay profile was fitted by deconvolution with the instrumental response (see Methods), yielding three kinetic components with *τ_X_* = 10 ns, τ_X*_ = 1 ns, and *τ_XX_* = 80 ps as shown in Figure 3b (top panel), which reports the lifetime values and respective relative weights. The relative weights of the *XX* and X* components are consistent with CsPbBr_3_ NCs of the same size,^[^ ^58^, ^59^ ^]^ with the latter being nearly negligible, confirming the essentially total release of secondary carriers outside the primary NC and the impossibility of reaching (and thus charging) another NC in dilute solution. The average excitonic population extracted from the time‐resolved trace of the colloidal solution according to ref. [57] is N≈1, which agrees with recent results on NCs of the same size under the same X‐ray excitation.^[^ ^26^ ^]^

More importantly, NC‐MSNs show a significantly more intense ultrafast contribution, driven by a marked increase in the X* component (≈1000% increase, Figure 3b), with a slight increase in the *XX* contribution. This is consistent with the increased density of photoelectrons in NC‐MSNs, which, in addition to exciting the luminescence of NCs, promotes the formation of charged states. As a result, the RL becomes more intense and faster, reaching an effective lifetime $\tau_{EFF}∼1\;ns$ in the highly loaded d = 200 nm particles (sample 200H, τ_*EFF*_ is defined as the weighted harmonic average of the decay contributions, see Methods). As a counterexample to this effect, the dynamics of the *d* = 350 nm NC‐MSNs (sample 350L) with lower RL and higher ^1^O_2_ generation rate show a relative weight of the X* component lower than the corresponding *d* = 200 nm NC‐MSNs (Figure 3b). Finally, it is instructive to evaluate the overall trend of the relative performance in pulsed detection resulting from the RL intensification (*I_RL_*) and the concomitant reduction of τ_*EFF*_ in NC‐MSN under X‐ray excitation by the so‐called pulse quality,^[^ ^63^ ^]^ expressed as $P\;∼\sqrt{\frac{I_{RL}}{\tau_{EFF}}}$. Considering *I_RL_* = 1 for colloidal NCs and the relative RL intensities shown in Figure 2b,c, an improvement in *P* of up to 9 times from the colloidal NCs to the 200L NC‐MSNs is obtained (inset of Figure 3c), which is valuable for fast timing applications.^[^ ^12^, ^38^ ^]^

**Figure 4:**
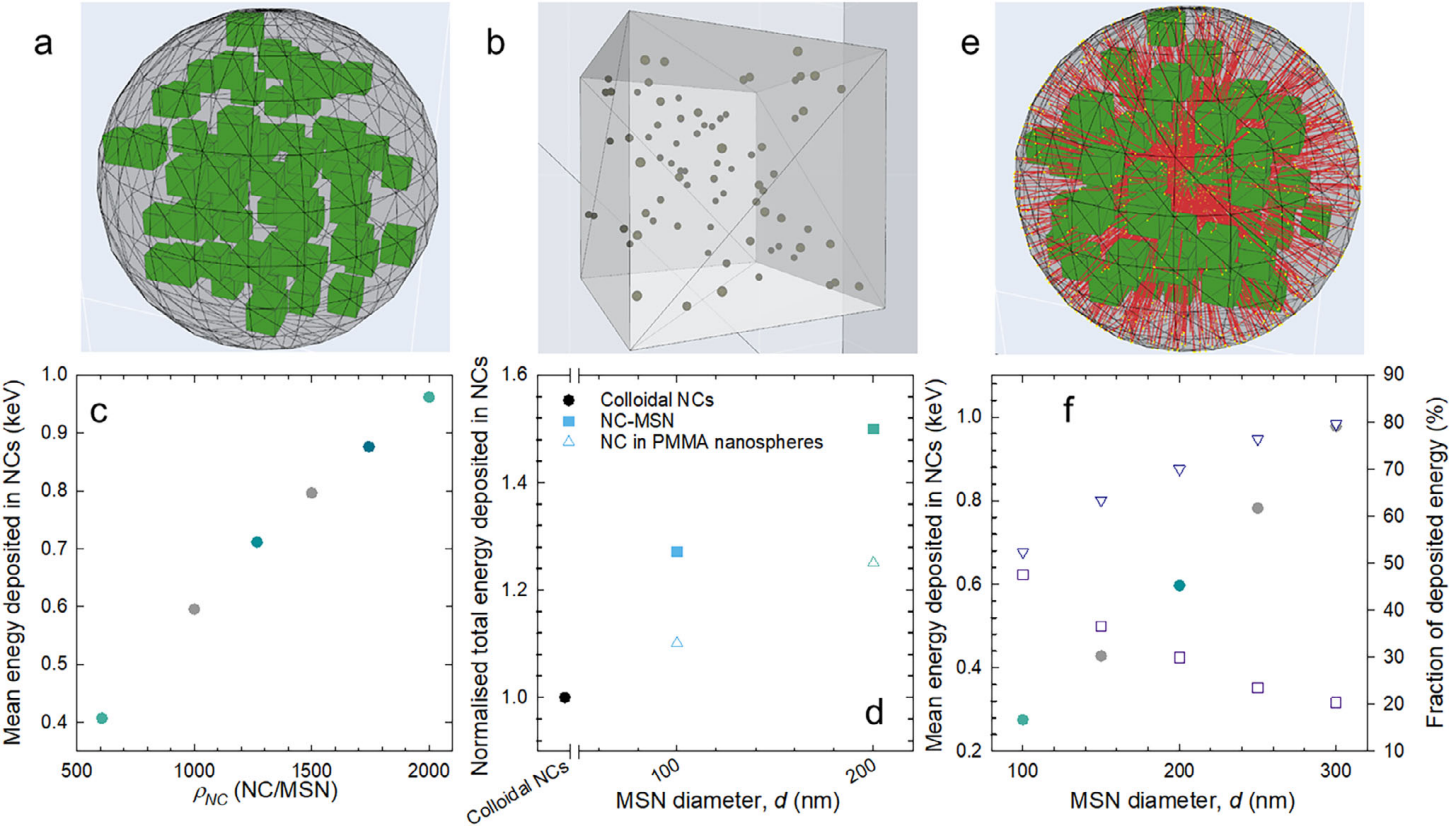
Monte Carlo simulations of energy deposition. a) Schematic representation of a single NC‐MSN containing 77 CsPbBr_3_ NCs, generated using code A. b) Simulation of a polyacrylate cube (side length: 3 µm) embedding 77 NC‐MSNs, performed using code B. c) Mean energy deposited per X‐ray in CsPbBr_3_ NCs as a function of increasing NC density (*ρ_NC_*) within a single NC‐MSN, simulated with code A. d) Total energy deposited in CsPbBr_3_ NCs, expressed as a percentage relative to the colloidal NC case, as a function of increasing MSN diameter. Gray data points correspond to simulations in which SiO_2_ was replaced by polymethyl methacrylate. These simulations were conducted using code B. e) Geometry of a single NC‐MSN identical to that in panel (a), featuring an electron source located within a central NC. f) Mean energy deposited per electron in NC‐MSNs of varying diameters (circles). Squares indicate the fraction of energy deposited in the NC where the electrons are initially generated, while triangles represent the fraction deposited in surrounding NCs. Values are normalized to the total energy deposited in all other NCs.

With the aim of gaining a deeper understanding of the energy deposition processes in CsPbBr_3_ NCs confined within MSNs, a simulation was finally developed using Geant4 (version 11.1.2), a toolkit for simulating radiation‐matter interactions. Two simulation codes were implemented to model different geometric configurations that replicate the experimental conditions: a single NC‐MSN system (code A) and a larger ensemble containing multiple NC‐MSNs (code B). In code A, the geometry consists of a single SiO_2_ nanosphere with a diameter of 200 nm, filled with randomly distributed, nonoverlapping CsPbBr_3_ NCs (each with an edge length of 8 nm, **Figure** **4**a). Code B, by contrast, models a system in which multiple NC‐MSNs (as defined in code A) are randomly distributed—without overlap—within a 3 µm‐sided polyacrylate cube (Figure 4b). The cube size was selected to balance computational cost and the number of NC‐MSNs. To accurately simulate electromagnetic interactions, the *G4EmStandardPhysics_option4* physics list was used, chosen for its high precision in electron tracking and use of advanced low‐energy models. In both codes A and B, the radiation source was modelled as an X‐ray beam (10^7^ photons) with a continuous energy spectrum ranging from 0 to 20 keV, matching the experimental RL conditions.

Code A was used to investigate how energy deposition in CsPbBr_3_ NCs varies with increasing NC density (*ρ_NC_*) within a single NC‐MSN. As shown in Figure 4c, the energy deposited per X‐ray increases linearly with *ρ_NC_*. To further examine how the spatial arrangement of NCs within MSNs affects total energy deposition, code B was used to simulate three distinct configurations of NC ensembles embedded in a polyacrylate matrix: *i*) 5929 randomly dispersed individual CsPbBr_3_ NCs; *ii*) 77 NC‐MSNs (100 nm diameter), each containing 77 NCs; *iii*) 10 NC‐MSNs (200 nm diameter), each containing 616 NCs. Finally, to isolate the respective contributions of geometric confinement and material composition to the observed enhancement in energy deposition, a conceptual simulation (*gedankenexperiment*) was performed in which the SiO_2_ in the MSNs was replaced with polymethyl methacrylate. The results (Figure 4d) show an increase in energy deposition when NCs are confined within MSNs. This enhancement appears to arise equally from geometric confinement and the higher atomic number of the MSN material. We point out that the lack of quantitative agreement between the experimental RL enhancement and the simulated energy deposition in Figure 4d is likely to be due to scintillation effects, which are not captured by the current simulation and will be addressed in a dedicated study due to their complexity. Next, we investigated the energy deposition from secondary electrons in NCs embedded within MSNs of varying diameters using code A. For this simulation, a source of 10^6^ electrons was randomly generated within a single NC located at the center of the MSN (Figure 4e). This setup was further used to evaluate how the deposited energy is distributed between the NC where the electrons originate and the surrounding NCs.

As illustrated in Figure 4f, the total energy deposited within the system increases with the diameter of the MSN. In smaller NC‐MSNs, a substantial portion – ≈50% – of the energy is deposited in the NC where the electrons are initially generated. As the MSN size increases, the relative contribution of the source NC decreases, indicating that while overall energy retention becomes stronger, the energy deposition is more evenly distributed among surrounding NCs. Finally, we reproduced the same geometry and analysis, with the only difference being that the electrons were generated in an NC located near the surface edge of the MSN, as shown in Figure S12a (Supporting Information). Figure S12b,c (Supporting Information) show, respectively, the energy deposited in the MSN and the fraction absorbed by the first NC and by the remaining NCs. The differences observed in this configuration, compared to electron generation in an NC at the center of the MSN, can be attributed to geometrical effects: the proximity to the surface allows a significant fraction of electrons to escape the sphere, depositing little energy. To achieve the same amount of deposited energy as in the central NC case, it is necessary to double the radius of MSNs. This can be clearly seen by comparing Figure S12b (Supporting Information) with Figure 4d.

## 3 Conclusion

In conclusion, this study demonstrates that the local densification of CsPbBr_3_ NCs within porous silica mesospheres enables a remarkable enhancement of scintillation intensity up to 40 times greater than that of equivalent colloidal NC solutions. This improvement is attributed to the increased efficiency in converting secondary charges generated by the interaction with ionizing radiation. The results highlight how the spatial organization of NCs into high‐density domains can overcome the intrinsic limitations of traditional nanocomposites, paving the way for the development of high‐performance “metasolids” scintillators.

## Conflict of Interest

The authors declare no conflict of interest.

## Supporting information



Supporting Information

## Data Availability

The data that support the findings of this study are available from the corresponding author upon reasonable request.
