# Supplementary material for: Harnessing Self‐Sensitized Scintillation by Supramolecular Engineering of CsPbBr3 Nanocrystals in Dense Mesoporous Template Nanospheres
Source: Adv Mater. 2025 Oct 15;38(4):e13469. doi: 10.1002/adma.202513469 (PMC12810599; doi:10.1002/adma.202513469)
Supplement: Supplementary file 1 — Supporting Information [file ADMA-38-e13469-s001.docx]

# Supporting Information

**Harnessing self-sensitized scintillation by supramolecular engineering of CsPbBr_3_ nanocrystals in dense mesoporous template nanospheres**

Xiaohe Zhou^1^, Matteo L. Zaffalon^1,2^, Emanuele Mazzola^3^, Andrea Fratelli^1,2^, Francesco Carulli^1^, Chenger Wang^1^, Mengda He^4^, Francesco Bruni^1,2^, Saptarshi Chakraborty^1^, Leonardo Poletti^5^, Francesca Rossi^5^, Luca Gironi^2,3*^, Francesco Meinardi^1^, Liang Li^6^ and Sergio Brovelli^∗1,2^

^1^Dipartimento di Scienza dei Materiali, Università degli Studi di Milano-Bicocca, Via R. Cozzi 55, 20125, Milano, Italy

^2^INFN - Sezione di Milano - Bicocca, Milano I-20126, Italy

^3^Dipartimento di Fisica, Università degli Studi di Milano-Bicocca, Piazza della Scienza, 20125 Milan, Italy

^4^School of Environmental Science and Engineering, Shanghai Jiao Tong University, Shanghai 200240, China

^5^IMEM-CNR, Parco Area delle Scienze 37/A - 43100 Parma, Italy

^6^Macao Institute of Materials Science and Engineering (MIMSE), Macau University of Science and Technology, Taipa 999078, Macao, China

Email: Sergio Brovelli - [sergio.brovelli@unimib.it](mailto:sergio.brovelli@unimib.it), [luca.gironi@unimib.it](mailto:luca.gironi@unimib.it)

^∗^ Corresponding author

# Methods

**Synthesis of CsPbBr_3_ nanocrystals.**

*Chemicals.* Cesium carbonate *(*Cs_2_CO_3_, 99.9%, Sigma-Aldrich), diisooctylphosphinic acid (DOPA, 90%, Sigma-Aldrich), lead bromide (PbBr_2_, 99.999%, Sigma-Aldrich), trioctylphosphine oxide (TOPO, 90%, Strem), n-octane (97%, Acros Organics), hexane (96%, Scharlau), Oleic acid (OA, 90%, Sigma-Aldrich), lecithin (≥97%, from soy, Roth); mesitylene (99%, Thermo Scientific Chemicals); phosphorus(V) oxychloride (99%, Sigma-Aldrich); 2-aminoethan-1-ol (≥99.0%, Sigma-Aldrich); acetic acid (>99.8%, Sigma-Aldrich); triethylamine (99%, Sigma-Aldrich); 2-octyl-1-dodecanol (97%, Sigma-Aldrich).

*Stock solutions.* PbBr_2_-TOPO stock solution was prepared by dissolving 367 mg PbBr_2_ and 2.15 g TOPO in 5 ml n-octane at 120 °C, followed by cooling down and dilution with 20 ml hexane. Cs-DOPA stock solution was prepared by reacting 100 mg Cs_2_CO_3_ with 1 ml DOPA in 2 ml n-octane at 120 °C, followed by cooling down and dilution with 27 ml hexane. For mixed-halide NCs, ZnCl_2_-TOPO and ZnI_2_-TOPO stock solution for anion exchange were prepared by dissolving 136 mg ZnCl_2_ or 319 mg ZnI_2_ with 1.933 g TOPO in 2.5 ml octane at 120 °C, followed by cooling down and dilution with 7.5 ml hexane. 2-ammonioethyl 2-octyl-1-dodecyl phosphate (C_8_C_12_-PEA) ligand was synthesized as reported in Ref. *Nature* 626, 542–548 (2024).

*Synthesis.* NCs were synthesized according to the modified PbBr_2_-TOPO method in Ref. *Science* 377, 1406-1412 (2022). A 25 ml flask was loaded with 3 ml hexane and 2 ml PbBr_2_-TOPO stock solution and stirred at 1200 rpm. Next, 1 ml Cs-DOPA stock solution was injected, and the solution was stirred for 3 minutes before the ligand was added (C_8_C_12_-PEA, 0.26 ml, 0.1M in mesitylene). The crude solution was rotary evaporated at room temperature until 5-7ml left, washed with acetone (0.4 eq.), centrifuged at 12100 rpm, and redispersed in n-octane. To obtain mixed-halide NCs, ZnCl_2_-TOPO or ZnI_2_-TOPO stock solutions were added (equimolar to the PbBr_2_) before the washing, followed by stirring for a few minutes.

**Synthesis CsPbBr_3_-SiO_2_ MSN.**

*Chemicals.* Cesium bromide (CsBr, 99.5%, Aladdin), lead bromide (PbBr_2_, 99%, Aladdin), Potassium carbonate (K_2_CO_3_, 99%, Aladdin), cetyltrimethylammonium bromide (CTAB, 95%, Aladdin) and tetraethylorthosilicate (TEOS, 95%, Aladdin), sodium hydroxide (NaOH, 98%, Aladdin), cesium carbonate (Cs_2_CO_3_, 99.9%, Aladdin), 1-octadecene (ODE, 90%, Aladdin), oleylamine (OAm, 90%, Aladdin), oleic acid (OA, 90%, Aldrich), F127 (EO_106_PO_60_EO_106_, AR, Macklin), ethanol (99.5%, Sinopharm Chemical Reagent), methyl acetate (98%, Sinopharm Chemical Reagent), toluene (99.5%, Sinopharm Chemical Reagent), meso- tetra(4-sulfonatophenyl) porphyrin (H_2_TPPS^4–^) (Combi-Blocks).

*Preparation of MSNs.* MSNs were synthesized by the following procedure: cetyltrimethylammonium bromide (CTAB, 1.00 g) and F127 (100mg) was first dissolved in 480 mL of ultrapure water. Then, 3.0 mL of a NaOH (2M) solution was added and stirred in a water bath at 80 °C for 30min. 5.0 mL TEOS was then dropped into the above solution. The mixture was stirred for 2 hours and the solid was collected by centrifugation, washed with ultrapure water, and dried at 80°C. To prepare the final MSNs the synthesized solid was calcined for 6 hours in an oxidizing atmosphere at 550 °C to remove the template. By changing the volume of the NaOH (2M) solution to adjust the size of MSNs, the MSNs of 100nm and 350nm diameter correspond to 2.1mL and 3.9mL of NaOH solution, respectively.

*NC-MSNs synthesis.* The preparation of NC-MSNs was performed following a previously optimized procedure. Specifically, for medium NC loading, 0.6 mmol of salt precursors (127.69 mg of CsBr and 220.20 mg of PbBr 2 ) were dissolved in 50 mL ultrapure water, sonicate for 5 min and stir continuously at 80 °C for 30 min until clear. Then around 1050 mg of MSNs (pre-dispersed in 20 mL ultrapure water) (the mass ratio of NC precursors: MSNs=1:3) was added into the above solution. The mixture is stirred continuously at 80°C until dry. The collected mixture was ground and calcined at 600°C temperature for 30 min with a heating rate of 5 °C/min in a muffle furnace under air atmosphere. After cooling to room temperature, the sample was ground and washed with ultrapure water several times to remove external NC or other salts. Finally, the washed sample was obtained by centrifugation and drying at 80 °C. By changing the mass ratio of NC precursors to MSNs to adjust the loading of NC, low loading and high loading correspond to NC precursors : MSNs= 0.5:3 and 2:3, respectively.

*Morphological and elementary characterization.* The powder X-ray diffraction (XRD) patterns of samples

were performed by a Bruker D8 Advance X-ray Diffractometer at 40 kV and 30 mA using Cu Kα radiation (λ = 1.5406 Å). TEM/STEM imaging and EDX spectroscopy were performed in a JEOL JEM-2200FS microscope, operated at 200 kV, equipped with an high-angle annular dark field detector for Z-contrast imaging and an Oxford Xplore detector for compositional analysis. The particles were deposited by drop-casting on Cu grids with ultrathin carbon support film.

*Optical spectroscopy.* Optical absorption measurements were measured in octane with an Agilent Cary 60 UV–Vis spectrophotometer. PL measurements were performed by exciting the samples with a 405 nm pulsed diode laser (Edinburgh Inst. EPL 405, 40 ps pulse width), and collected with a TM-C10083CA Hamamatsu Mini-Spectrometer. PLQY for every sample was obtained by comparison with a standard with the same absorbance at the excitation energy. Time-resolved PL were carried out using the same 405nm source (Edinburgh Inst. EPL 405, 40 ps pulse width) in PL measurement; the emitted light was collected with a phototube coupled to a Cornerstone 260 1/4 m VIS-NIR Monochromator (ORIEL) and a time-correlated single-photon counting unit (time resolution ∼400 ps). Ultrafast transient absorption spectroscopy measurements were performed on Ultrafast Systems Helios TA spectrometer. The laser source was a 10 W Ytterbium amplified laser operated at 1.875 kHz producing ~260 fs pulses at 1030 nm and coupled with an independently tunable optical parametric amplifier from the same supplier that produced the excitation pulses at 3.1 eV (400 nm). After passing the pump beam through a synchronous chopper phase-locked to the pulse train (0.938 kHz, blocking every other pump pulse), the pump fluence on the sample was modulated using a variable ND filter. The probe beam was a white light supercontinuum.

*RL measurements* Unfiltered X-rays were produced using a Philips PW2274 X-ray tube with a tungsten target, equipped with a beryllium window and operated at 20 kV to produce a continuous X-ray spectrum through bremsstrahlung. The scintillation light was detected using a liquid-nitrogen-cooled, back-illuminated, UV-enhanced CCD detector (Jobin Yvon Symphony II), coupled to a monochromator (Jobin Yvon Triax 180) with a 100 lines/mm grating. CL maps were acquired using an electron beam current of 300 pA and an accelerating voltage of 5 kV.

*LY measurements* Light yield values were determined by comparing the integrated RL intensity under 20 kV X-ray excitation (⟨E⟩ ~ 7 keV) with identical experimental conditions for a 1 wt% octane solution of CsPbBr_3_ NCs and NC-MSNs placed in a 5 mm long crucible and a commercial EJ-276D plastic scintillator (LY = 8600 photons/MeV) of the same size and geometry used as a reference. In both cases, the sample size was chosen to ensure complete attenuation of the excitation beam.

*Time resolved scintillation measurements* The time-resolved RL was measured using a pulsed X-ray source consisting of a 405 nm ~70-ps pulsed laser hitting the photocathode of an X-ray tube (N5084, Hamamatsu) set at 40 kV. The emitted scintillation light was collected using an FLS980 spectrometer (Edinburgh Instruments) coupled to a PicoHarp 300 hybrid photomultiplier tube operating in TCSPC mode. The RL decay curves were analyzed using a least-squares fitting approach with the following formula, which accounts for the convolution with the instrument response function (IRF):

$$F\left( t \right)=IRF\left( t \right)\otimes\left( H\left( t-t_{0} \right)\cdot\left[ \sum_{i=1}^{2} a_{i}\cdot e^{-t/\tau_{i}} \right] \right)+C$$

where $t_{0}$​ corresponds to the start of the emission process, *C* is the electronic background noise floor, and *H* is the Heaviside function. The experimental IRF was well described by a Gaussian profile (FWHM = 120 ps), and the weight of each component ($w_{i}$​) was calculated as the integral of each convoluted function over the entire time window. The average lifetime was calculated using the re-normalized ratio of all components $\tau_{i}$according to:

$\tau_{eff}=\left( \frac{\tau_{1}}{w_{1n}}+\frac{\tau_{2}}{w_{2n}} \right)^{-1}$, $w_{in}=\frac{w_{i}}{w_{1}+w_{2}}$

*Singlet Oxygen Generation Measurement.* The optical probe SOSG has been purchased from Thermo Fisher. The SOSG powder has been diluted in a 1:10 solution of dimethyl sulfoxide (DMSO) and PBS, which has been used to disperse the CsPbBr_3_-SiO_2_ with a concentration of 4 mg/mLin water. The intensity of the SOSG fluorescence, which is directly proportional to the concentration of singlet oxygen in the environment, has been monitored during the X-ray exposure under continuous-wavelength laser light excitation at 473 nm.

*Evaluation of ROS production rate.* A 4 mL PBS solution containing the same concentration of SOSG used in the ROS production experiment (8.3×10^-5^ M) was prepared and 0.5 mg of meso-tetra(4-sulfonatophenyl) porphyrin (H_2_TPPS^4–^), an efficient photosensitizer for singlet oxygen generation, were added and the final solution and kept under stirring in dark condition until the complete dissolution of H_2_TPPS^4–^. We performed the complete oxidation of SOSG via the photo-sensitizer approach rather than the radio-sensitizer to avoid exposing the sample under extremely high X-ray dose rate, which may result in undesired sample degradation. The solution was maintained under stirring and exposed to 405 nm while the SOSG PL was monitored using an in-situ fiber with 473 nm excitation. UV exposition was maintained until no further increment of SOSG PL was observed, which indicates the complete oxidation of the SOSG in the solution (**Figure S6**). The ratio between the PL collected at the end (corresponding to complete SOSG oxidation) and before UV irradiation (22.3 times higher) was used to evaluate the fraction of total SOSG moles which are oxidized and thus the ROS moles produced:

$$m_{ROS}(t)=\frac{{PL}_{SOSG}(t) 1}{{PL}_{SOSG} (0) 22.3} 8.3\times{10}^{-5}M\times V$$

Where PL_SOSG_ (t) is the PL of the SOSG evaluated after a specific exposure time t, 8.3 × 10^-5^ M is the molarity of the SOSG solution and V is the volume of the solution.

|  | Atomic Fraction (%) | | | | | | | | | | |
| --- | --- | --- | --- | --- | --- | --- | --- | --- | --- | --- | --- |
|  | 100nm | | 200nm L | | 200nm M | | 200nm H | | 350nm | | |
|  | Avg | SD | Avg | SD | Avg | SD | Avg | SD | Avg | SD |  |
| O | 65.21 | 1.32 | 62.58 | 1.11 | 61.32 | 1.17 | 60.43 | 1.62 | 63.93 | 1.45 |  |
| Si | 31.84 | 1.33 | 33.40 | 0.48 | 32.10 | 1.13 | 30.77 | 1.34 | 34.16 | 1.14 |  |
| Cl | 0.22 | 0.07 | 0.11 | 0.10 | 0.55 | 0.24 | 0.68 | 0.15 | 0.06 | 0.06 |  |
| K | 0.36 | 0.12 | 0.43 | 0.07 | 0.10 | 0.03 | 0.05 | 0.03 | 0.00 | 0.01 |  |
| Br | 1.43 | 0.46 | 2.15 | 0.33 | 3.52 | 0.83 | 4.64 | 0.27 | 1.07 | 0.34 |  |
| Cs | 0.45 | 0.12 | 0.68 | 0.13 | 1.25 | 0.29 | 1.83 | 0.11 | 0.41 | 0.11 |  |
| Pb | 0.49 | 0.13 | 0.64 | 0.11 | 1.17 | 0.24 | 1.59 | 0.09 | 0.37 | 0.09 |  |
| LHP | 2.37 | 0.69 | 3.48 | 0.56 | 5.93 | 1.35 | 8.06 | 0.42 | 1.85 | 0.53 |  |

**Table S1.** EDX analysis on NC-MSN sample set as atomic fraction, following the same color code as in main report.

| **Composition** | **Sample Form** | **Wt%** | **Thickness (mm)** | **Scintillation Time (ns)** | **LY**  **(ph MeV^-1^)** | **Ref.** |
| --- | --- | --- | --- | --- | --- | --- |
| CsPbBr_3_ NCs in Mesoporous Silica Spheres (200 nm, high loading) | Octane solution | 1 | -- | 1 | 40000 | This work |
| CsPbBr_3_@Cs_4_PbBr_6_ | Thin film | -- | -- | 3 | 6000 | ^[1]^ |
| CsPbBr_3_ NCs @ BaF_2_ | Heterostructure | -- | 84μm | 11 | 6300 | ^[2]^ |
| CsPbBr_3_ NCs in glass-ceramic matrix | Glass matrix | -- | 0.2-1mm | 27 | 4100 | ^[3]^ |
| CsPbBr_3_ NCs | Octane solution | 2.5 | 1mm | -- | 2300 | ^[4]^ |
| CsPbBr_3_/Cs_4_PbBr_6_ NCs | NC powders | -- | -- | -- | 3600 | ^[5]^ |
| CsPbBr_3_/Cs_4_PbBr_6_ NCs | NC powders | -- | -- | -- | 64000 | ^[6]^ |
| CsPbBr_3_/CsPb_2_Br_5_ NCs | Thin film | -- | 20-5μm | -- | 19200 | ^[7]^ |
| CsPbBr_3_/CsPb_2_Br_5_ NCs | Water solution | 0.2 | -- | 100s | ~3000 | ^[8]^ |
| CsPbBr_3_ NCs + dye | Solution | 2.5 | -- | -- | 16700 | ^[9]^ |
| CsPbBr_3_ NCs | NCs in anodized Al oxide | -- | 20-30μm | -- | 11100 | ^[10]^ |
| CsPbBr_3_ NSs | Thin film | -- | 4μm | -- | 21000 | ^[11]^ |
| CsPbBr_3_ NWs | NWs in anodized Al oxide | -- | 50μm | -- | 13200 | ^[12]^ |
| CsPbBr_3_ NCs | Film | -- | 2mm | -- | 24000 | ^[13]^ |
| CsPbBr_3_ NCs | Powders | -- | -- |  | 1100 | ^[14]^ |
| CsPbBr_3_:F NCs | Powders | -- | -- | -- | 8500 | ^[14]^ |
| CsPbBr_3_ NCs + dye | Solution | 40 | -- | 24.3 | ~8000 | ^[15]^ |
| CsPbBr_3_ NCs | Film | -- | 30μm | -- | 21000 | ^[16]^ |
| CsPbBr_3_ NCs/MOF | Powder coating | -- | 200μm | 36 | 24800 | ^[17]^ |
| ZnS(Ag)-CsPbBr_3_ | Powder Heterostructure | -- | -- | 36 | 40000 | ^[18]^ |
| CsPbBr_3_ NWs | Thin film | 4 | 800μm | -- | 3800 | ^[19]^ |
| CsPbBr_3_ NCs/MOF | Film | -- | -- | 35 | 13673 | ^[20]^ |

**Table S2:** Key physical properties and scintillation performance of significant CsPbBr_3_ based nanoscintillators

**Supporting References**

[1] F. Cao, D. Yu, W. Ma, X. Xu, B. Cai, Y. M. Yang, S. Liu, L. He, Y. Ke, S. Lan, K.-L. Choy, H. Zeng, ACS Nano 2020, 14, 5183.

[2] H. Yang, H. Li, R. Yuan, J. Chen, J. Zhao, S. Wang, Y. Liu, Q. Li, Z. Zhang, Journal of Materials Chemistry C 2021, 9, 7905.

[3] H. Zhang, Z. Yang, M. Zhou, L. Zhao, T. Jiang, H. Yang, X. Yu, J. Qiu, Y. Yang, X. Xu, Advanced Materials 2021, 33, 2102529.

[4] S. Cho, S. Kim, J. Kim, Y. Jo, I. Ryu, S. Hong, J.-J. Lee, S. Cha, E. B. Nam, S. U. Lee, S. K. Noh, H. Kim, J. Kwak, H. Im, Light: Science & Applications 2020, 9, 156.

[5] Z. Li, Q. Hu, Z. Tan, Y. Yang, M. Leng, X. Liu, C. Ge, G. Niu, J. Tang, ACS Applied Materials & Interfaces 2018, 10, 43915.

[6] Q. Xu, J. Wang, W. Shao, X. Ouyang, X. Wang, X. Zhang, Y. Guo, X. Ouyang, Nanoscale 2020, 12, 9727.

[7] V. Naresh, S. Singh, H. Soh, J. Lee, N. Lee, Materials Today Nano 2023, 23, 100364.

[8] H. Lian, W. Zhang, R. Zou, S. Gu, R. Kuang, Y. Zhu, X. Zhang, C.-G. Ma, J. Wang, Y. Li, Advanced Materials 2023, 35, 2304743.

[9] M. Liu, L. Huang, D. Yuan, Z. Li, Y. Teng, J. Zhang, S. Huang, B. Liu, ACS Appl. Nano Mater. 2023, 6, 370.

[10] H. Li, H. Yang, R. Yuan, Z. Sun, Y. Yang, J. Zhao, Q. Li, Z. Zhang, Advanced Optical Materials 2021, 9, 2101297.

[11] Y. Zhang, R. Sun, X. Ou, K. Fu, Q. Chen, Y. Ding, L.-J. Xu, L. Liu, Y. Han, A. V. Malko, X. Liu, H. Yang, O. M. Bakr, H. Liu, O. F. Mohammed, ACS Nano 2019, 13, 2520.

[12] H. Dierks, Z. Zhang, N. Lamers, J. Wallentin, Nano Res. 2022, 16, 1084.

[13] F. Maddalena, A. Xie, X. Y. Chin, R. Begum, M. E. Witkowski, M. Makowski, B. Mahler, W. Drozdowski, S. V. Springham, R. S. Rawat, N. Mathews, C. Dujardin, M. D. Birowosuto, C. Dang, J. Phys. Chem. C 2021, 125, 14082.

[14] M. L. Zaffalon, F. Cova, M. Liu, A. Cemmi, I. Di Sarcina, F. Rossi, F. Carulli, A. Erroi, C. Rodà, J. Perego, A. Comotti, M. Fasoli, F. Meinardi, L. Li, A. Vedda, S. Brovelli, Nature Photonics 2022, 16, 860.

[15] H. Yu, T. Chen, Z. Han, J. Fan, Q. Pei, ACS Appl. Nano Mater. 2022, 5, 14572.

[16] Z.-W. Lü, G.-X. Wei, H.-Q. Wang, Y. Guan, N. Jiang, Y.-Y. Liu, Z. Li, H. Qin, H.-Q. Liu, Nuclear Science and Techniques 2022, 33, 98.

[17] L. Gu, Z. Yang, J. Cui, Z. Feng, J. Yao, J. Song, ACS Nano April 14, 2025, 19.

[18] Z. Yang, J. Yao, L. Xu, W. Fan, J. Song, Z. Yang, J. Yao, L. Xu, W. Fan, J. Song, Nature Communications 2024 15:1 2024-10-14, 15.

[19] Z. Wang, S. Li, G. Ren, S. Yao, D. Zhu, J. Xie, J. Zhou, X. Xu, W. Huang, Y. Kuai, C. M. Wan, J. Ye, X. Xu, Z. Hu, ACS Photonics July 3, 2024, 11.

[20] J. Zhou, J. Zhou, Z. Wang, Z. Wang, Z. Shi, Z. Shi, X. Zhang, X. Zhang, L. Yang, L. Yang, Y. Jiang, Y. Jiang, Y. Kuai, Y. Kuai, Z. Hu, Z. Hu, S. Li, S. Li, Nanoscale 2025/03/24, 17.

# Estimation of CsPbBr_3_ Nanocrystal Content from EDX Atomic Fractions

To estimate the density *ρ_NC_* of CsPbBr_3_ NCs embedded within the MSN, we utilized the elemental atomic fractions obtained from energy-dispersive X-ray spectroscopy (EDX). The following procedure outlines the calculation based on compositional and structural assumptions:

The atomic fractions of Si, O, Cs, Pb, and Br, obtained from EDX, are denoted as *η*_Si_, *η*_O_, *η*_Cs_, *η*_Pb_, and *η*_Br_, respectively. The total atomic fraction satisfies:

*η*_Si_ + *η*_O_ + *η*_Cs_ + *η*_Pb_ + *η*_Br_ = 100%

To estimate the molar content of SiO_2_ and CsPbBr_3_, we account for the number of atoms per formula unit: three atoms for SiO_2_ and five atoms for CsPbBr_3_. The number of moles of each species is approximated as:

$$n_{SiO2}=\frac{\eta_{\mathrm{Si}}+\eta_{O}}{3}; n_{{CsPbBr}_{3}}=\frac{\eta_{\mathrm{Cs}}+\eta_{\mathrm{Pb}}+\eta_{\mathrm{Br}}}{5}$$

The molar fraction of CsPbBr_3_ NC in NC-MSN is given by:

$$\chi_{{SiO}_{2}}=\frac{n_{SiO2}}{n_{SiO2}+n_{{CsPbBr}_{3}}}; \chi_{{CsPbBr}_{3}}=\frac{n_{{CsPbBr}_{3}}}{n_{SiO2}+n_{{CsPbBr}_{3}}}$$

The mass contributions of each component are calculated using their respective molar masses:

- - *M*SiO_2_: 60*.*08 g*/*mol
  - *M*CsPbBr_3_: 528*.*1 g*/*mol

The total mass of the mixture is expressed as:

*m*total = *n*SiO_2_ *· M*SiO_2_ + *n*CsPbBr_3_ *· M*CsPbBr_3_

The mass of CsPbBr_3_ is then:

*m*CsPbBr_3_ = *x*CsPbBr_3_ *· m*total

The bulk density of CsPbBr_3_ is 4*.*86 g*/*cm^3^ , so the total volume of embedded NC is estimated as:

$$V{CsPbBr}_{3}=\frac{m_{{CsPbBr}_{3}}}{{4.86g/cm}^{3}}$$

Assuming monodisperse cubic CsPbBr_3_ nanocrystals with edge length of 8 nm, the volume of a single nanocrystal is:

*V*_NC_ = (8 *×* 10*^−^*^7^ cm)^3^ = 5*.*12 *×* 10*^−^*^19^ cm^3^

Finally, the total number of nanocrystals is estimated by:

$$\rho= \frac{V\mathrm{CsPbBr}_{3}}{V_{NC}}$$

**

**Figure S1.** Size distribution of NC inside MSN and size distribution of MSNs.

**Figure S2.** Representative XRD intensity of NC-MSN (colored line) with d=200 nm together with the pattern for cubic (red line) and orthorhombic CsPbBr_3_ (blue line).


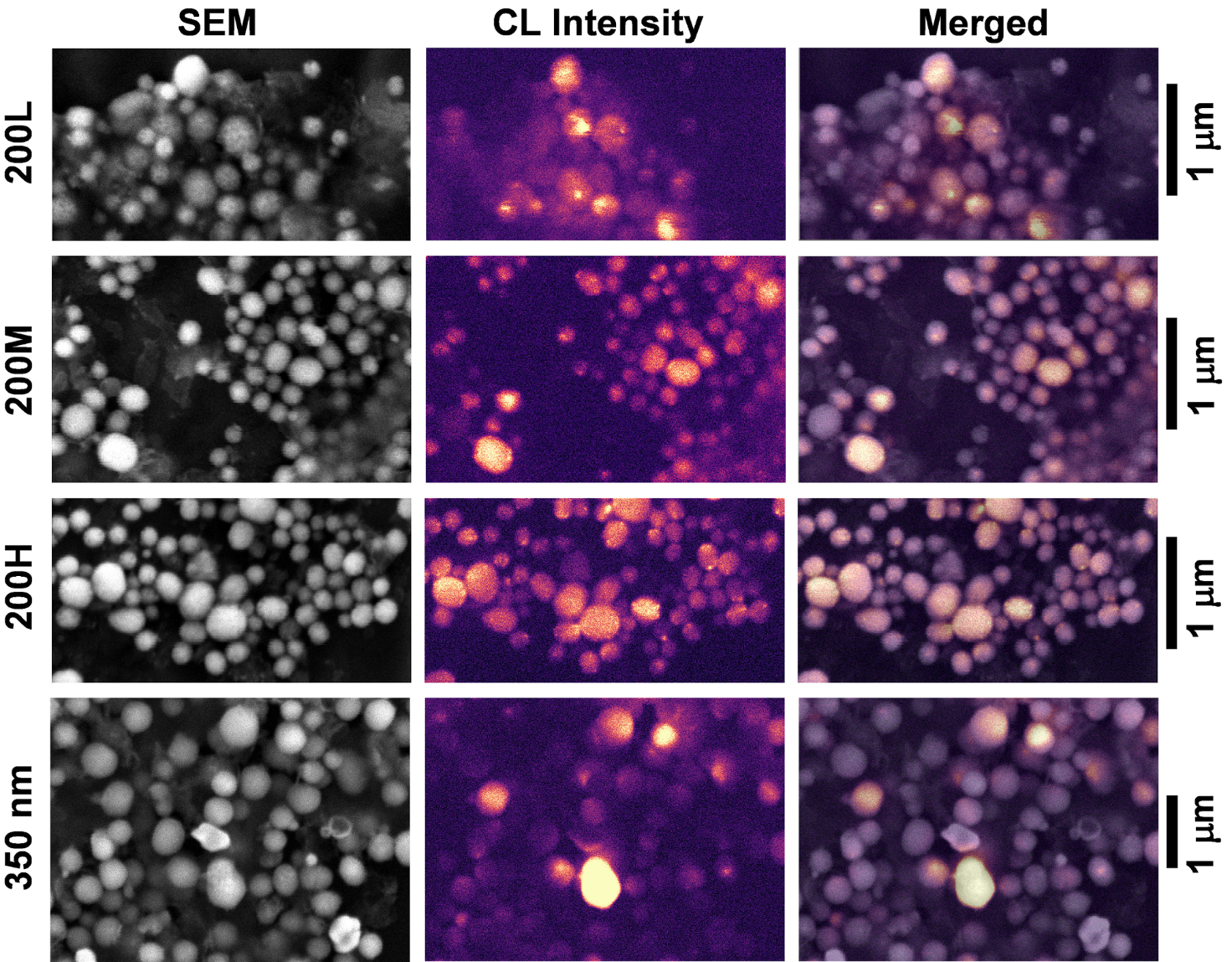


**Figure S3.** From left to right: SEM images, CL intensity maps and merged images of NC-MSNs samples with different diameters


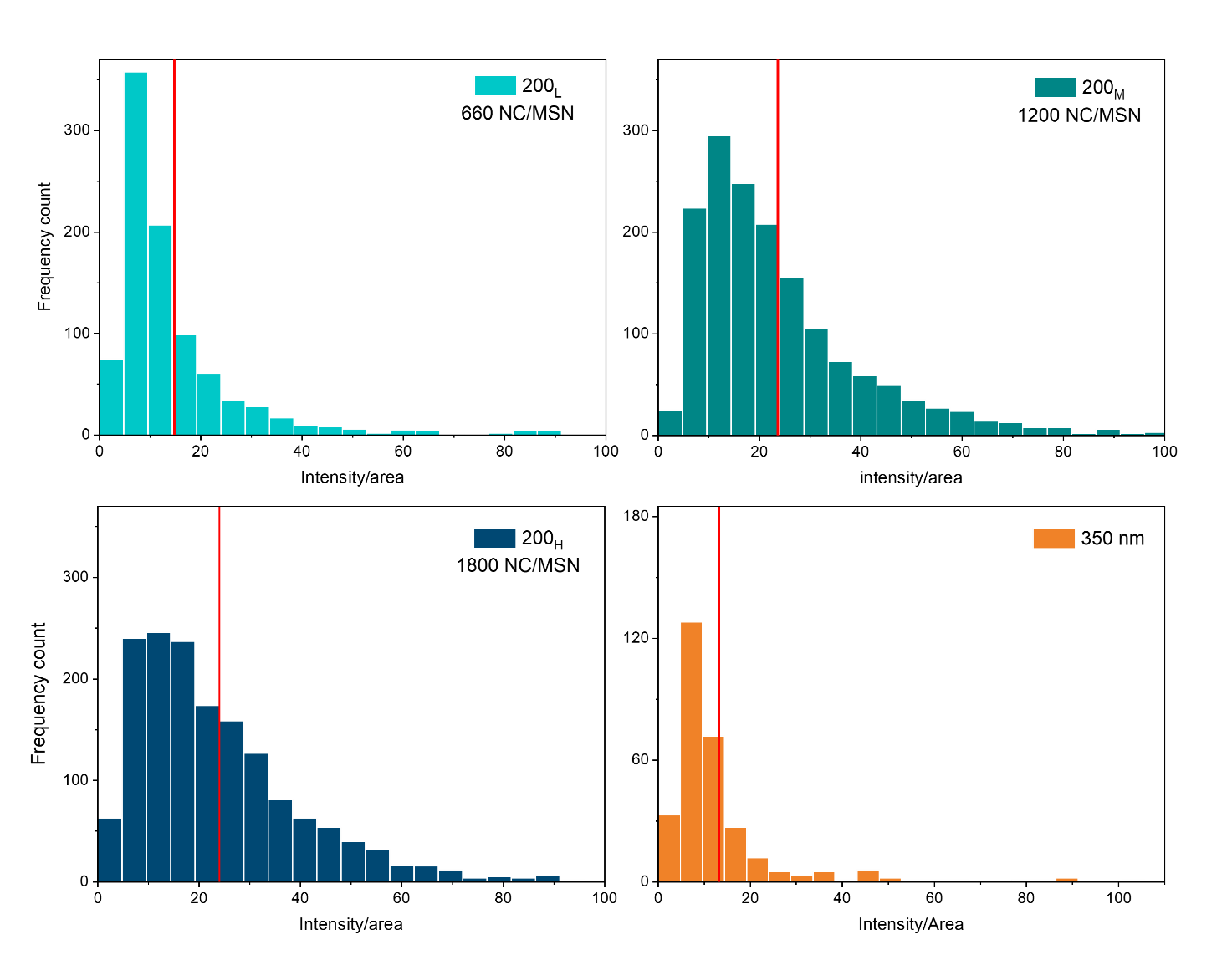


**Figure S4.** Statistical distribution of intensity per unit area obtained from CL maps. Red lines indicate the mean values. Those are 14.8 ± 0.5, 23.6 ± 0.4, 24.0 ± 0.4 and $13.2\pm0.8$ (a.u.), respectively.

Quantitative analyses were also performed to obtain a statistical distribution of the intensities emitted by individual spheres.

This analysis was carried out using ImageJ software, working on .tiff SE images and corresponding CL intensity maps. SE images were used to accurately define regions of interest (ROIs), each having a circular shape corresponding to the edge of a single MSN. The defined ROIs were then transferred onto the CL maps, allowing the measurement of both intensity and area for each MSN. Histograms showing the intensity per unit area of the analysed particles are reported in **Figure S4**.

The increase in luminescence observed when increasing the NC concentration from 660 to 1200 NCs/MSN is not further enhanced in the 200H sample. This behaviour is likely due to reabsorption effects occurring at high NC densities. Additional measurements varying the accelerating voltage and beam current may help to further investigate this phenomenon.


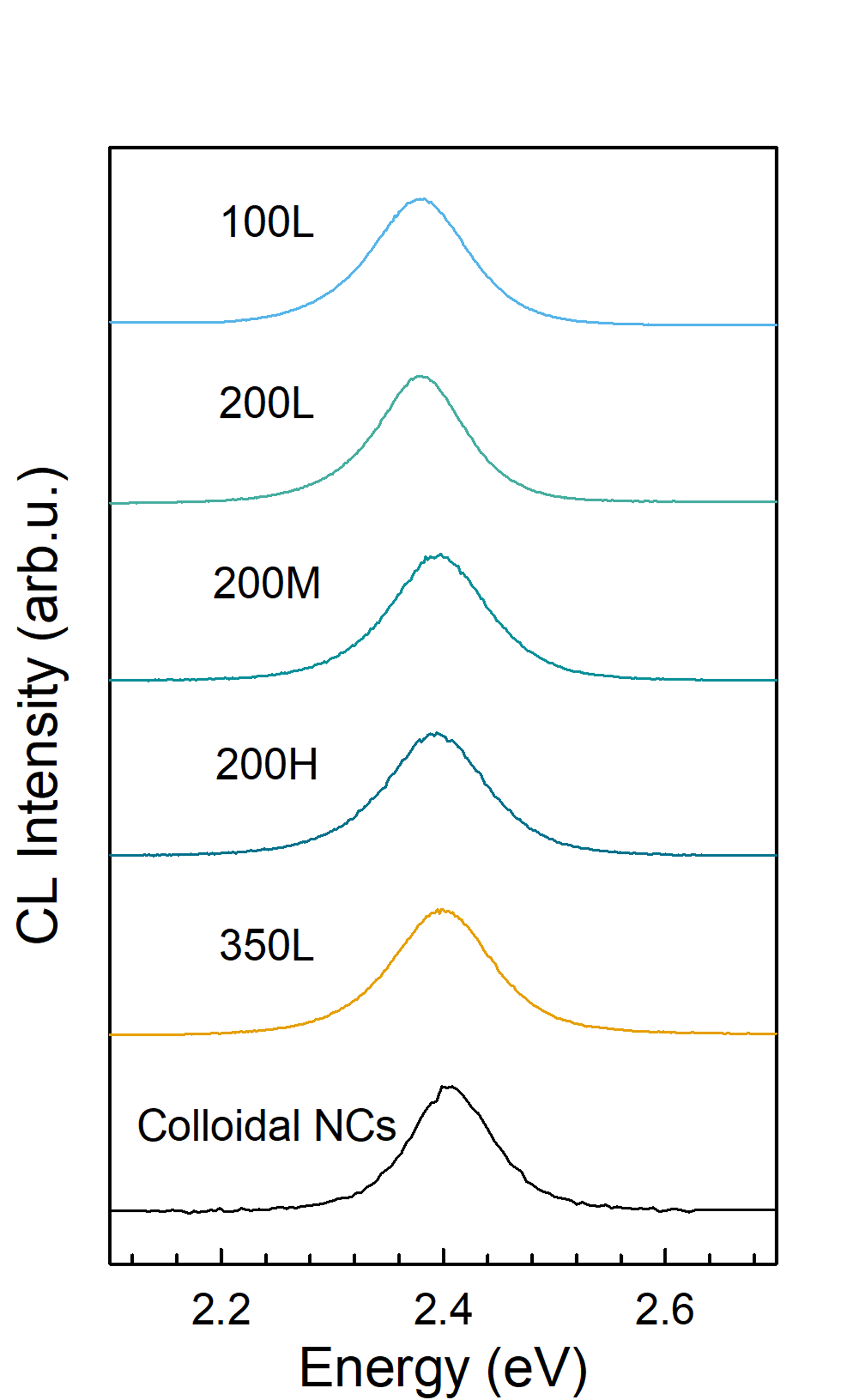


**Figure S5.** Cathodoluminescence spectra of CsPbBr3 NCs are related NC-MSNs.


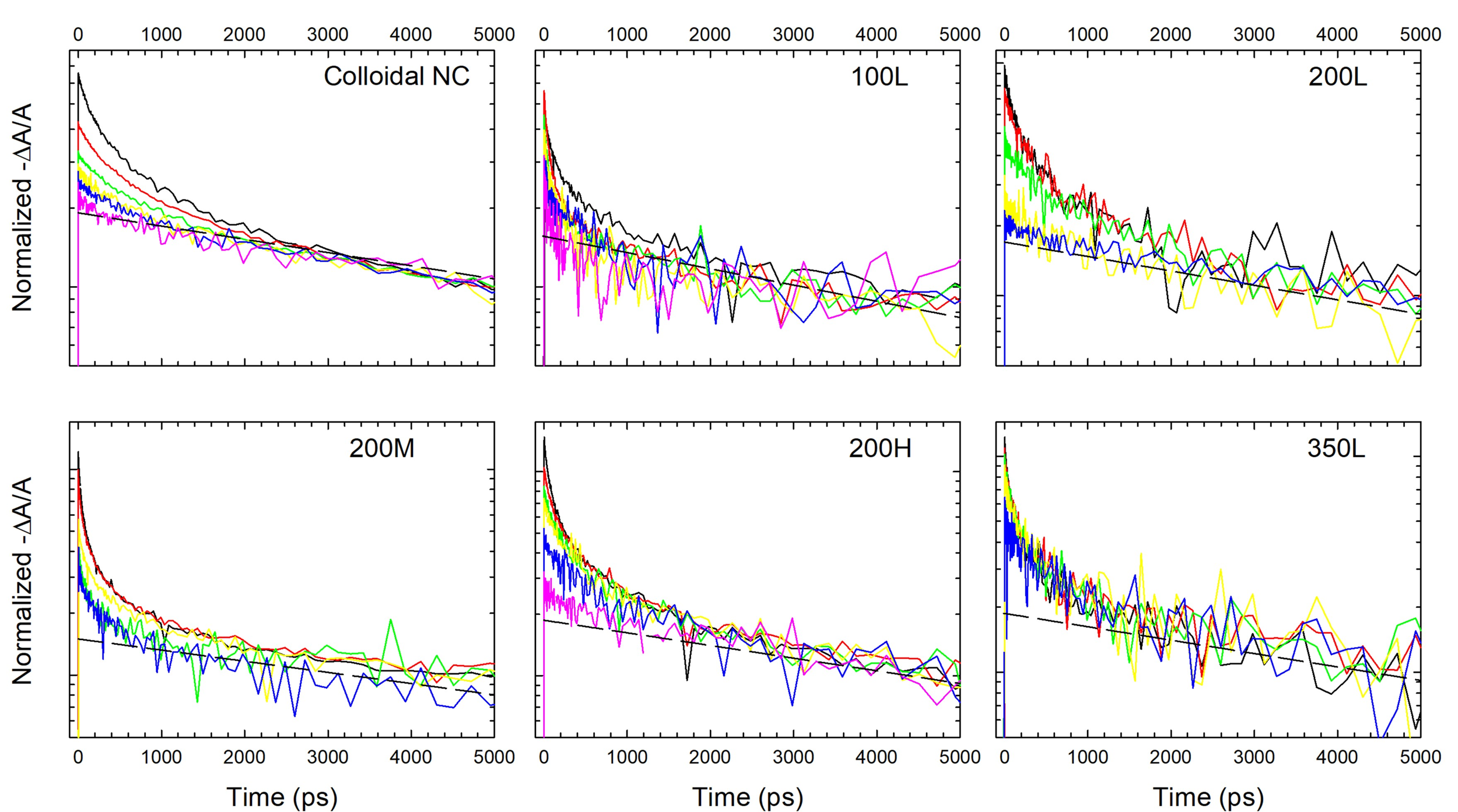


**Figure S6**. Transient absorption dynamics of colloidal NC and NC-MSNs under increasing excitation power (from <N> < 0.4 to <N> ~ 6) at 3.05 eV. The lines shade from blue to black as the fluence increases. Dashed line marked the single exciton contribution used in subtraction fitting method.


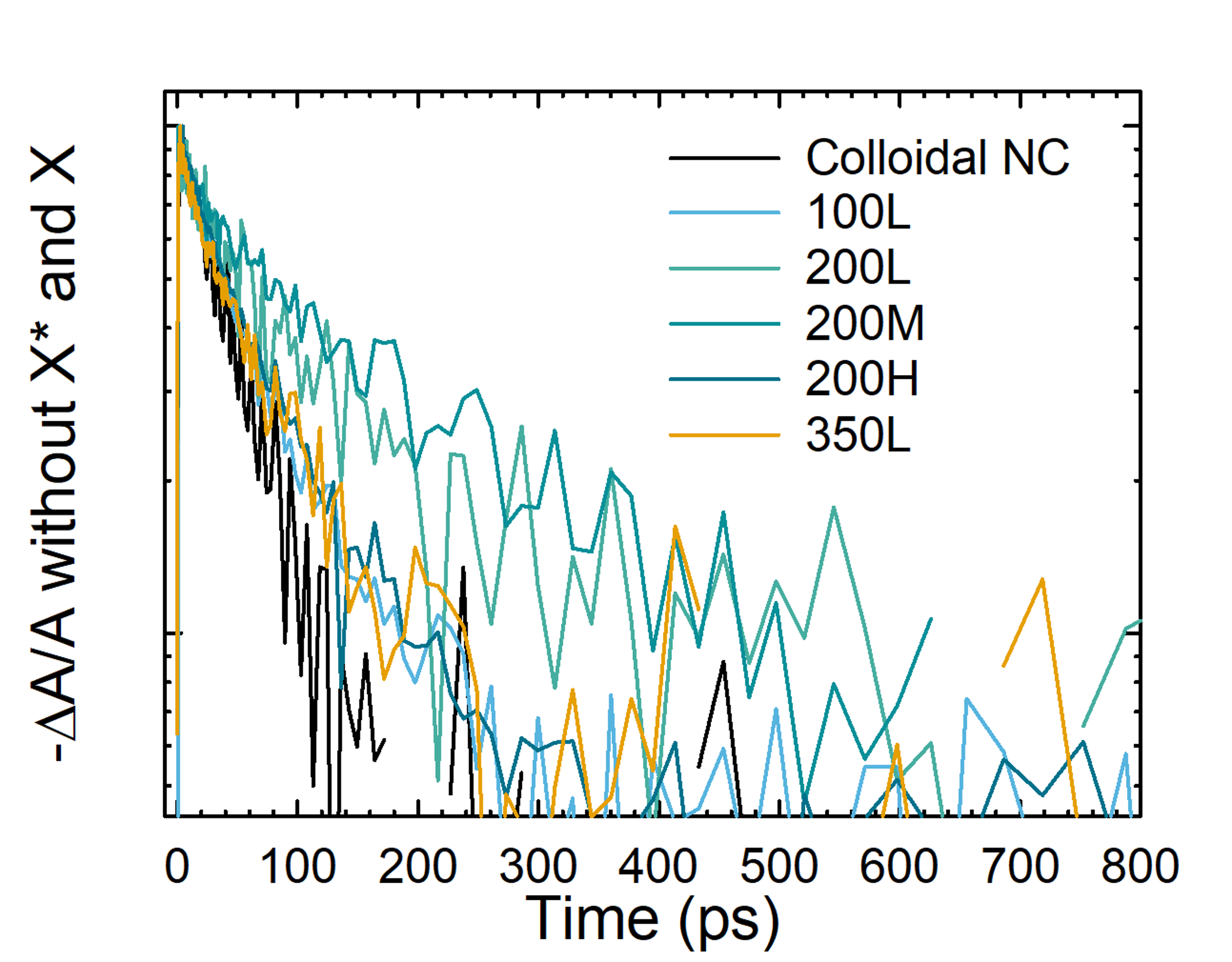


**Figure S7.** TA dynamics of *XX*. The fitting results of XX are used to calculate $\Phi_{\mathrm{XX}}$ in Figure 1e.


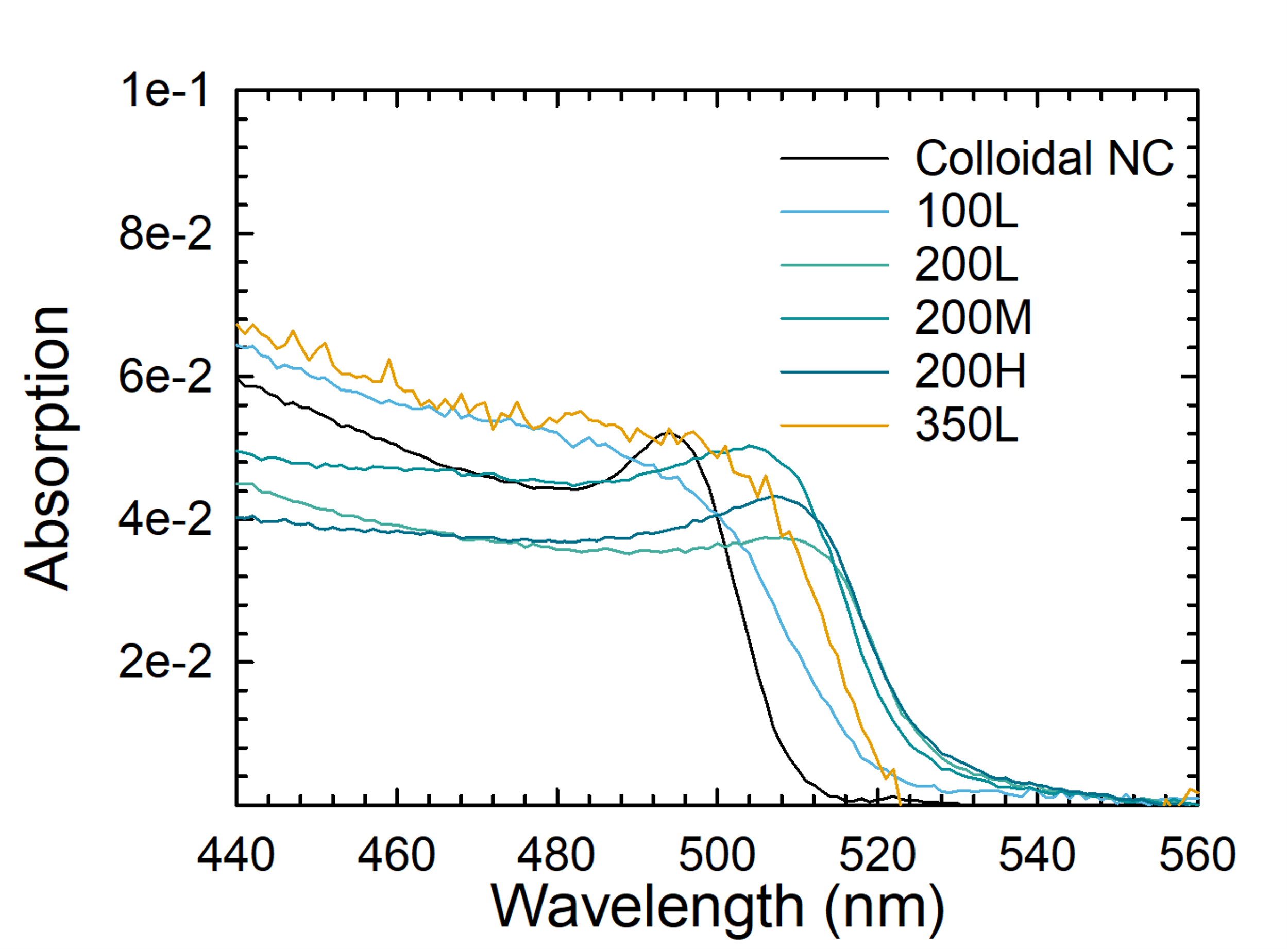


**Figure S8.** Absorption spectra of sample solutions for RL measurements with the same average concentration of CsPbBr_3_ NCs (implied by the same band edge absorption difference).

*
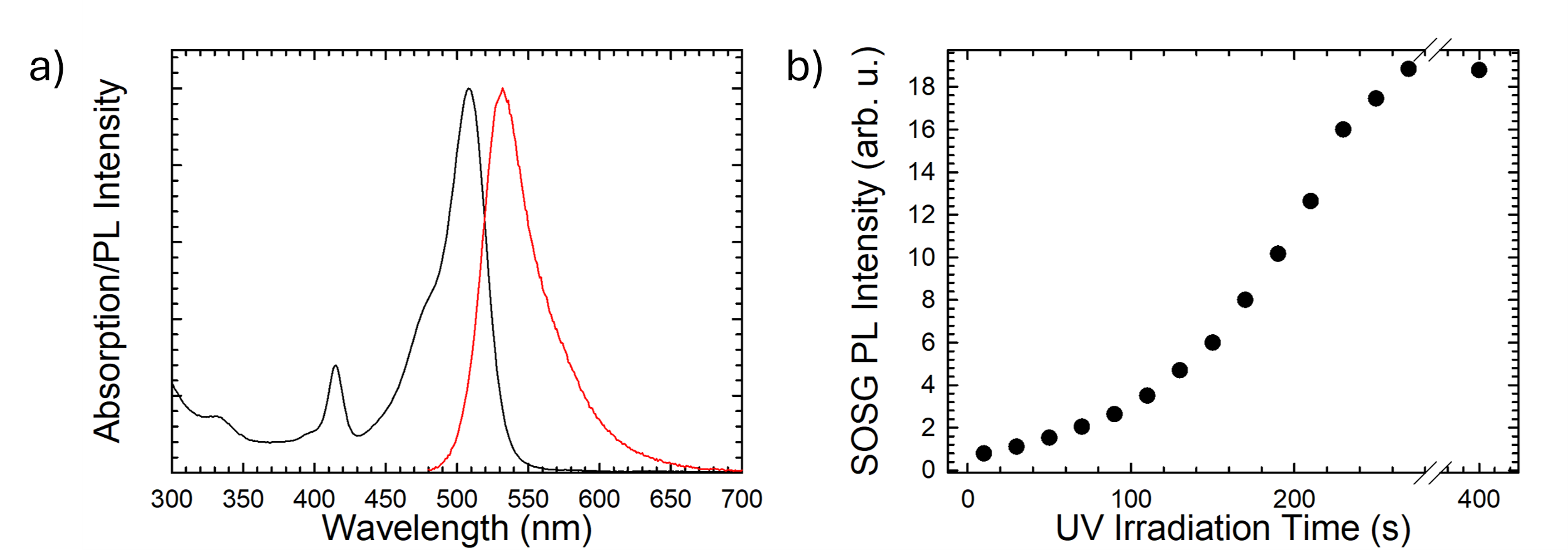
*

**Figure S9**. a) Absorption (black) and PL emission (red) spectra of SOSG. b) SOSG PL intensity growth using photo-sensitizing approach.

***
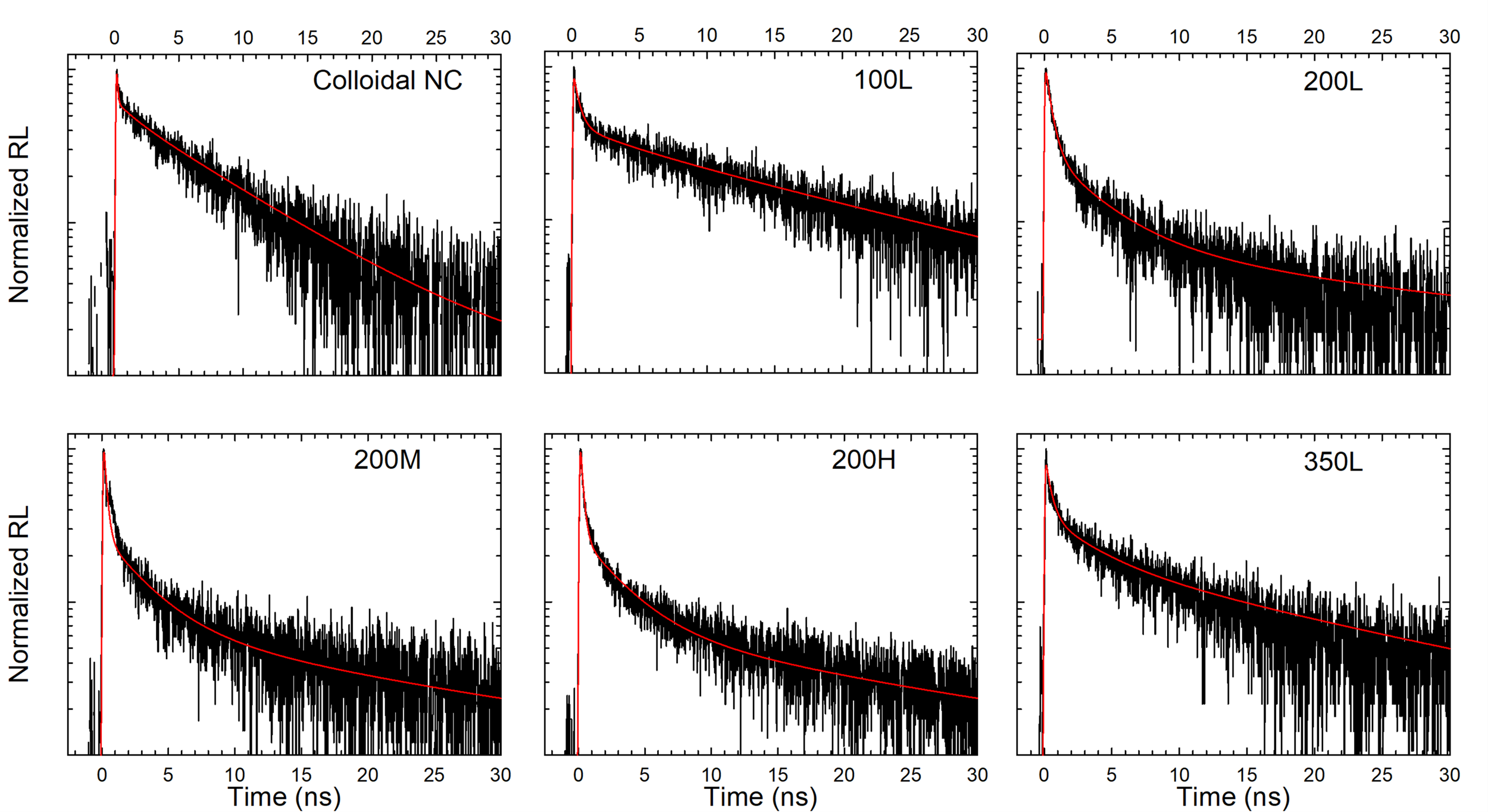
***

**Figure S10.** Time-resolved scintillation decay curves (black) of colloidal NC and NC-MSNs with their corresponding fit lines (red).

***
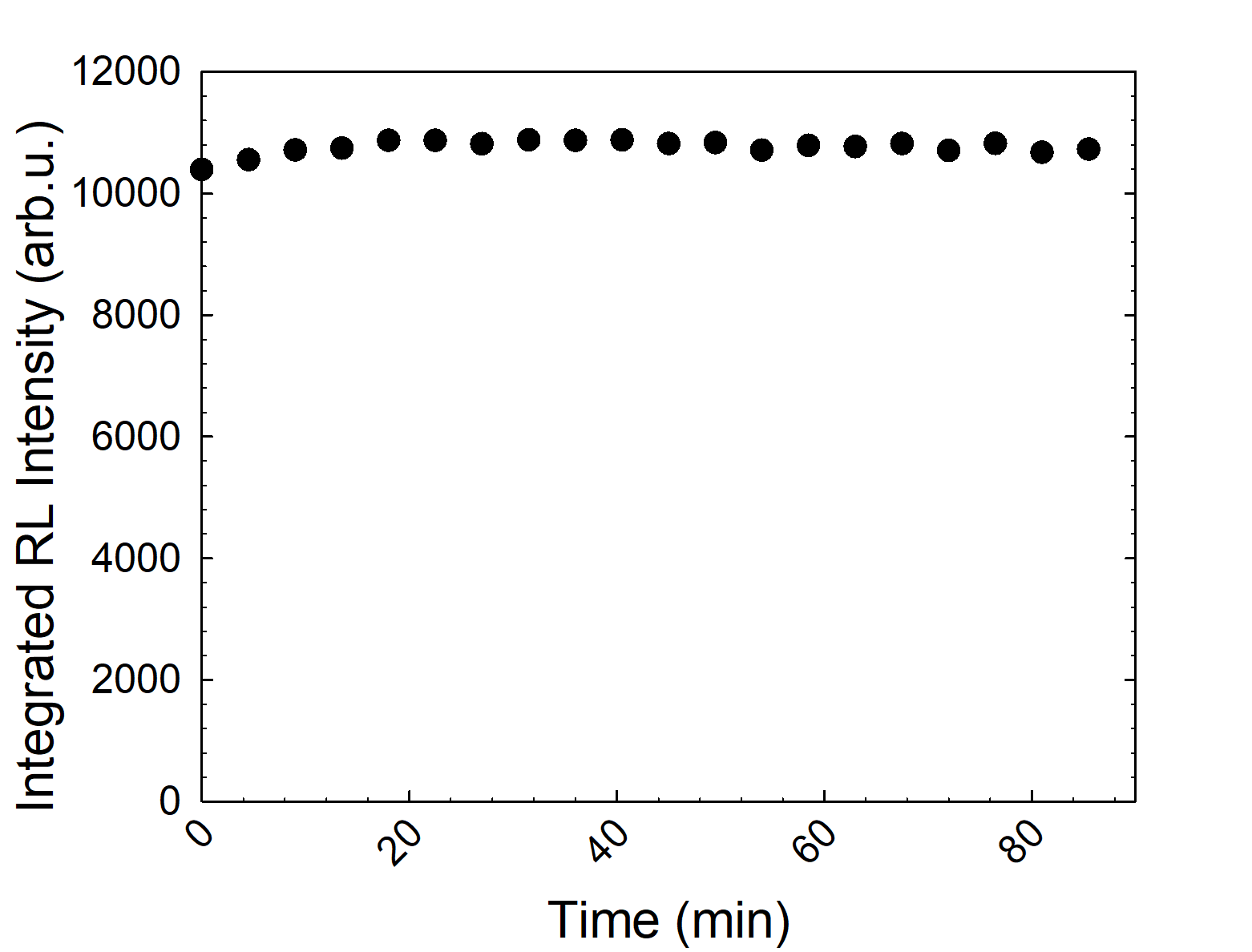
***

**Figure S11.** Integrated radioluminescence intensity of of NC-MSN measured for up to one hour of continuous X-ray irradiation at 20 kV, I = 20 mA corresponding to total absorbed doses of and 8.4 × 10^2^ Gy (calibration performed using crystalline quartz as the absorbing medium).


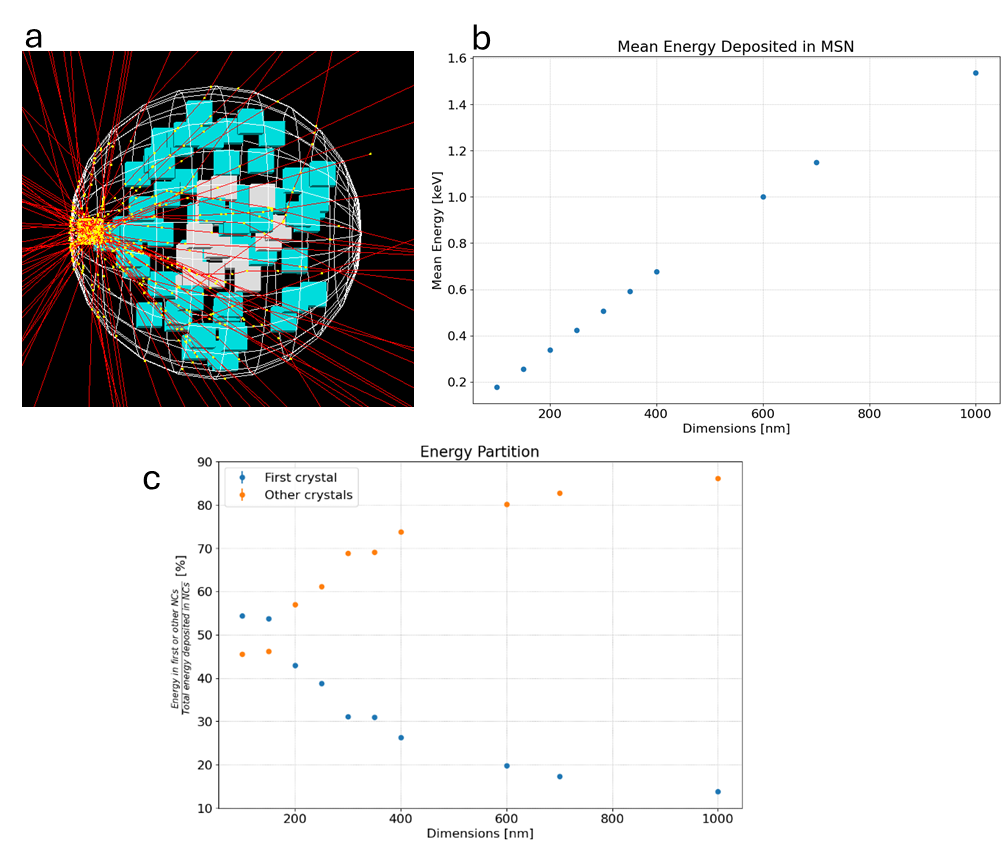


**Figure S12. a**, Simulated SiO_2_ NC-MSN with a NCs number density of 1.45x10^17^ cm^-3^ and with cubic CsPbBr_3_ NCs of side of 8 nm. Differently from Figure 4a, the electrons are generated inside a NC close to the surface of the MSN. **b**, Mean energy deposited by each electron in the NC-MSN system as a function of increasing MSN diameter. **c**, Percentage of the energy deposited in the NC where the electrons are generated (blue dots) and the other NC in the MSN (orange dots), normalized over the total energy deposited in all NCs.

***
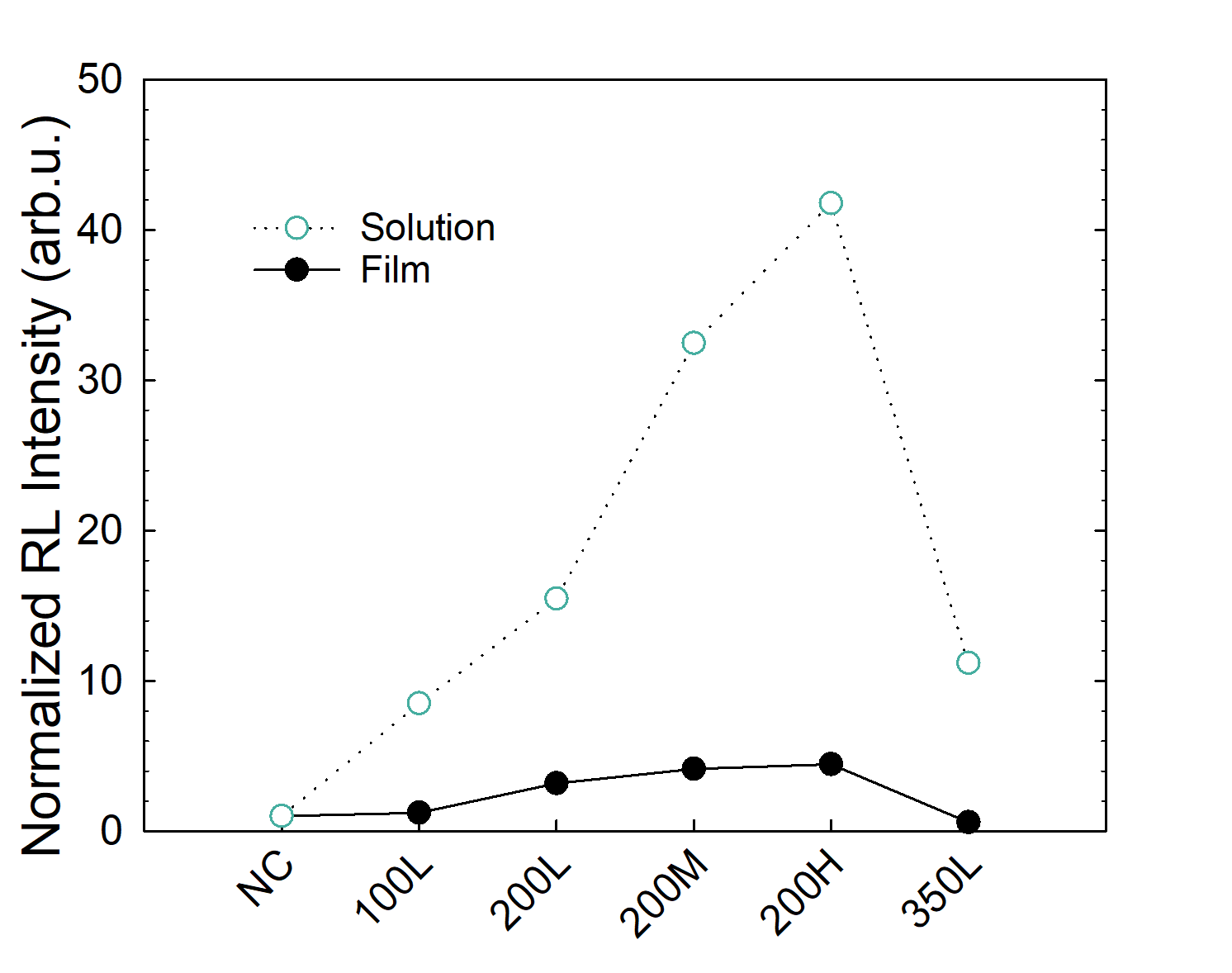
***

**Figure S13.** Comparison of the RL intensity trends (normalized to the respective value for colloidal NCs) of the solutions (hollow circles) and films (black dots) of the entire sample set, which contains the same amount of CsPbBr_3_ in each sample. Consistent with densification being the main factor in the RL trend, the film samples exhibit similar behavior, with a slight enhancement likely due to the interaction between SiO_2_ and X-rays.

***
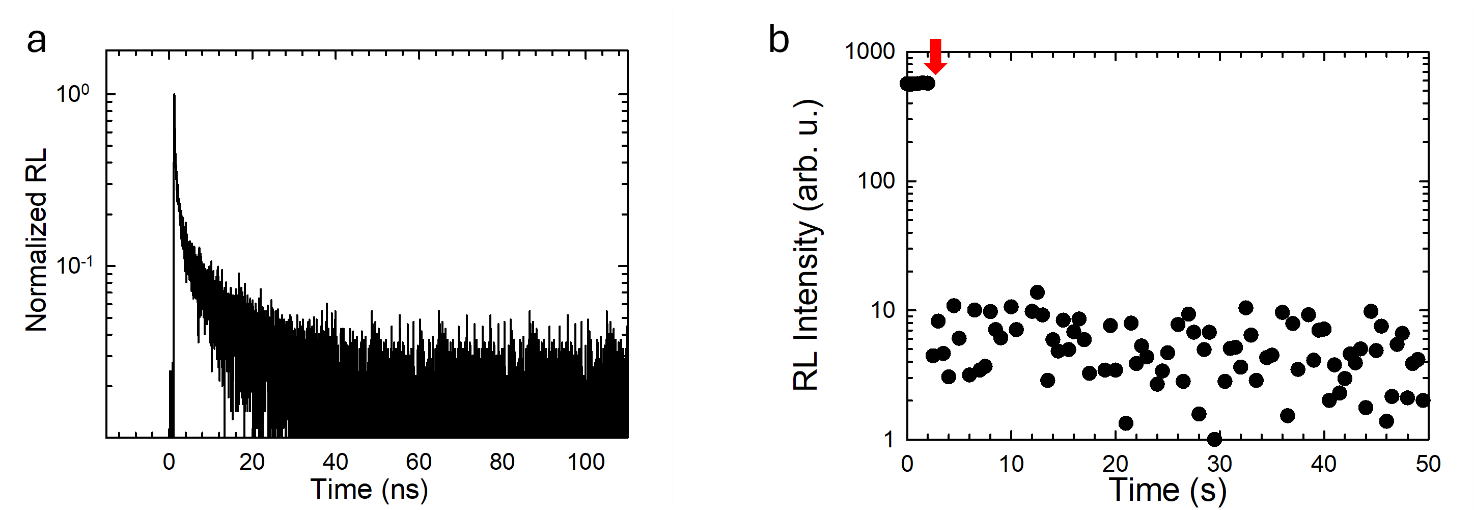
***

**Figure S14. a,** Time-resolved RL curve of sample 200L extending to over 100 ns, which shows complete decay of the RL signal within the first 50ns. **b**, Afterglow measurements at room temperature by collecting RL over time after 10 minutes of irradiation to check the presence of delayed emission from stable traps. No residual light was detected.
